# Negative Regulation of Active Zone Assembly by a Newly Identified SR Protein Kinase

**DOI:** 10.1371/journal.pbio.1000193

**Published:** 2009-09-22

**Authors:** Ervin L. Johnson, Richard D. Fetter, Graeme W. Davis

**Affiliations:** Department of Biochemistry and Biophysics, University of California San Francisco, San Francisco, California, United States of America; Technion Faculty of Medicine, Israel

## Abstract

Presynaptic, electron-dense, cytoplasmic protrusions such as the T-bar (*Drosophila*) or ribbon (vertebrates) are believed to facilitate vesicle movement to the active zone (AZ) of synapses throughout the nervous system. The molecular composition of these structures including the T-bar and ribbon are largely unknown, as are the mechanisms that specify their synapse-specific assembly and distribution. In a large-scale, forward genetic screen, we have identified a mutation termed *air traffic controller* (*atc*) that causes T-bar–like protein aggregates to form abnormally in motoneuron axons. This mutation disrupts a gene that encodes for a serine-arginine protein kinase (SRPK79D). This mutant phenotype is specific to SRPK79D and is not secondary to impaired kinesin-dependent axonal transport. The *srpk79D* gene is neuronally expressed, and transgenic rescue experiments are consistent with SRPK79D kinase activity being necessary in neurons. The SRPK79D protein colocalizes with the T-bar-associated protein Bruchpilot (Brp) in both the axon and synapse. We propose that SRPK79D is a novel T-bar-associated protein kinase that represses T-bar assembly in peripheral axons, and that SRPK79D-dependent repression must be relieved to facilitate site-specific AZ assembly. Consistent with this model, overexpression of SRPK79D disrupts AZ-specific Brp organization and significantly impairs presynaptic neurotransmitter release. These data identify a novel AZ-associated protein kinase and reveal a new mechanism of negative regulation involved in AZ assembly. This mechanism could contribute to the speed and specificity with which AZs are assembled throughout the nervous system.

## Abstract 2

A neuronal serine-arginine protein kinase that localizes to the presynaptic active zone is required for kinase-dependent repression of active zone assembly.

## Author Summary

Neurons communicate with each other through electrochemical impulses transmitted primarily at specialized intercellular junctions termed synapses. At each synapse, the primary site of synaptic vesicle fusion occurs at the active zone, an electron-dense presynaptic membrane with associated fibrillary matrix. Many active zones also possess one or more electron-dense cytosolic projections that are believed to facilitate vesicle mobilization to the active zone membrane and are required for normal synaptic transmission. These electron-dense projections are referred to as T-bars in *Drosophila* or ribbons in vertebrates. The molecular composition of these structures remains poorly characterized, and very little is known about how these structures are specifically assembled and stabilized at the presynaptic membrane. Here, we identify in *Drosophila* a neuronally expressed serine-arginine kinase called SRPK79D that localizes to the presynaptic active zone and that through its kinase activity appears to repress T-bar formation within peripheral axons. Our study thus provides evidence for kinase-dependent repression of active zone assembly, with implications for the development and growth of synaptic connections throughout the nervous system.

## Introduction

The majority of stimulus-dependent synaptic vesicle fusion occurs at presynaptic specializations called active zones (AZs). Ultrastructurally, AZs consist of at least two components; 1) a presynaptic membrane of high electron density, reflecting the presence of proteins such as Ca^2+^ channels, t-SNAREs, and cell adhesion molecules and 2) a fibrillary cytomatrix (CAZ) that includes cytoskeletal elements, scaffolding proteins, and AZ-specific molecules such as Piccolo/Aczonin, Bassoon, Unc-13/Dunc-13/Munc-13, RIM, and ELKS/Brp/ERC [1]. Many synapses that are characterized by a high release probability also include an electron-dense cytosolic projection that is believed to facilitate synaptic vesicle movement to the AZ. These projections are referred to as ribbons in the mammalian retina, dense bodies at the mammalian neuromuscular junction (NMJ), and T-bars at the *Drosophila* NMJ [1]–[3]. To date, five proteins have been found within the presynaptic ribbon at synapses in the vertebrate retina, including Piccolo, Kif3A, RIM, CtBP1, and RIBEYE/CtBP2 [1]–[3].

In *Drosophila*, there are no clear homologs of RIBEYE or Piccolo, and it remains unknown whether RIM or Kif3A associate with the *Drosophila* T-bar. The protein currently known to localize at the T-bar is the *Drosophila* homolog of ELKS/ERC, called Bruchpilot (Brp) [4],[5]. Recently, it was demonstrated that mutations in the *brp* gene eliminate T-bars and severely impair synaptic vesicle release, consistent with the conclusion that T-bars and Brp are essential components of the presynaptic AZ [4],[5].

T-bars and ribbons are large, macromolecular structures. In *Drosophila*, T-bars are first assembled at late embryonic stages as the nascent neuromuscular synapse begins to mature [6]–[8]. The appearance of T-bars and T-bar-associated antigens correlates with the ability of the neuromuscular junction to support larval movement. T-bars are formed only at the presynaptic face of the AZ and are not found at other sites, implying the existence of mechanisms that ensure site-specific assembly of these large, cytoplasmic structures. However, virtually nothing is known about how T-bar and ribbon structures are assembled and positioned at the AZ.

One model for AZ formation that could be extended to ribbon/T-bar assembly is based upon the existence of transport vesicles that contain AZ components, including calcium channels as well as the Piccolo and Bassoon proteins. It has been suggested that these transport vesicles fuse at sites of nascent synapse formation to deliver protein constituents of the AZ in a site-specific manner [9]–[11]. Although transport vesicles have not been isolated in *Drosophila* motoneurons, it was recently demonstrated that mutation of a Kinesin 3 (*immaculate connections*; *imac*) prevents the transport of synaptic vesicle proteins to the developing synapse, and in this mutant background, both AZ and T-bar formation are significantly impaired [8]. These data suggest that a critical component of AZ and T-bar assembly is contributed by Imac-dependent axonal transport. Although transport vesicles could represent a mechanism to deliver transmembrane and membrane-associated proteins to the AZ, there presumably exist other mechanisms to control the site-specific assembly of cytoplasmic proteins into a T-bar.

Here, we describe a previously uncharacterized gene in *Drosophila* that encodes a serine-threonine kinase that we have termed *serine-arginine protein kinase at 79D* (*srpk79D*). The SRPK79D protein is a member of the serine-arginine protein kinase family previously shown to be involved in mRNA splicing and processing [12]. This gene was identified in a large-scale forward genetic screen for genes involved in the development, maturation, and stabilization of the *Drosophila* NMJ. In this study, we present evidence that SRPK79D is a T-bar-associated protein kinase that is necessary to prevent premature T-bar assembly in peripheral axons. We also present evidence that SRPK79D activity must be overcome within the NMJ for normal AZ assembly and neurotransmission. As such, our data identify a new T-bar-associated antigen and indicate that synapse-specific assembly of the presynaptic T-bar may be achieved, in part, through suppression of T-bar assembly at nonsynaptic sites including the axon.

## Results

### *srpk79D* Loss of Function Causes Bruchpilot Accumulation in Larval Nerves

**Figure 1:**
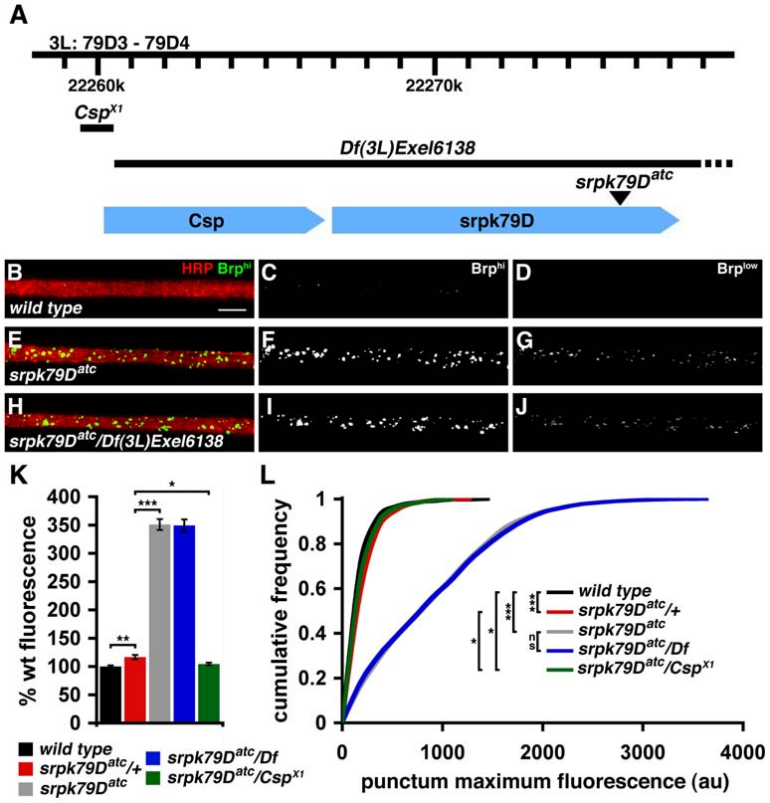
Brp accumulates in *srpk79D* mutant nerves. (A) The *srpk79D* gene region is shown, including the *srpk79D^atc^* transposon insertion site and deleted regions in mutants used for genetic analyses (black bars). Gene loci for *srpk79D* and the adjacent *Csp* gene are shown in blue. (B–J) Immunofluorescence images of larval nerves demonstrating large Brp accumulations in *srpk79D* loss-of-function mutants. Each image shows a section of a single larval nerve, photographed at the same relative position approximately 100 µm from where the nerve exits the CNS. Each nerve contains approximately 85 total axons, including approximately 35 motor axons. Images are shown at two exposures. Images in (B, E, and H) and (C, F, and I) were taken at an exposure in which small, infrequent anti-Brp puncta could be resolved in wild-type nerves. This resulted in overexposure of the Brp puncta in srpk79D mutant nerves. Images (D, G, and J) are identical to (B, E, and H, and C, F, and I) except taken at a lower exposure such that no puncta are found in wild type, and the puncta intensities are not saturated in the *srpk79D* mutant. (K) Total Brp fluorescence integrated over the nerve area is dramatically increased with *srpk79D* disruption, whereas loss of *Csp* does not increase nerve Brp levels. Each bar graph represents data collected from a total of 30 nerves from 12 different larvae. (L) Cumulative frequency plots of individual Brp punctum fluorescence intensities are shifted toward larger values with *srpk79D* loss of function (gray and blue lines representing *srpk79D^atc^* and *srpk79D^atc^/Df*, respectively, are shifted to the far right whereas other genotypes are clustered to the left). Each curve represents data collected from a total of 30 nerves from 12 different larvae. Sample size for wild type, *srpk79D^atc^*/+, *srpk79D^atc^*, *srpk79D^atc^/Df*, and *srpk79D^atc^/Csp^X1^* = 479, 1,715, 3,387, 3,718, and 2,714, respectively. Significance is indicated according to the following: * = *p*<0.05, ** = *p*<0.01, *** = *p*<0.001, and ns = not significant; Student *t*-test. Scale bar indicates 10 µm. Error bars indicate ±SEM. au = arbitrary units; Brp = anti-Bruchpilot; HRP = anti-horseradish peroxidase.

In an ongoing screen to identify genes involved in the formation and stabilization of the *Drosophila* NMJ, we identified a P-element insertion (P10036) in which the peripheral nerves contain numerous large accumulations of the AZ associated protein Brp (Figure 1E–1G). These large, aberrant Brp accumulations ranged from roughly spherical to grossly elongated in appearance (Figure 1E–1G). In wild-type animals, by contrast, axons within the peripheral nerves showed virtually no anti-Brp staining and the Brp puncta that did appear in these axons were small and spherical in appearance (Figure 1B–1D).

This phenotype is very unusual, based upon the results of our ongoing genetic screen. In this forward genetic screen, we have analyzed over 2,000 independent transposon insertion lines, including PiggyBac lines on chromosomes 2 and 3 from the Exelixis collection and an independent collection of P{GAL4} lines [13]. In each mutant background, we have stained three to five larvae with anti-Brp and anti-Discs Large (Dlg) antibodies and examined both the peripheral nerves and the neuromuscular synapse for defects. P10036 is the only mutation identified to date that causes the observed accumulation of anti-Brp staining in peripheral axons. The P10036 transposon resides within an intron of the previously uncharacterized gene *CG11489*, which resides at chromosomal position 79D and is predicted to encode a member of the SRPK family (Figure 1A and see below). Due to the dramatic effect on Bruchpilot (German for *crash pilot*) protein accumulation in peripheral axons, we named this mutant *air traffic controller* (*atc*), and we refer to P10036 as *srpk79D^atc^* throughout this article.

We next developed quantitative measures of the axonal Brp accumulations to further characterize and analyze the *srpk79D^atc^* mutant phenotype (see Materials and Methods). In all cases, genetic controls were dissected, processed, stained, and imaged identically and in parallel with *srpk79D^atc^* mutants. We found a statistically significant increase in total nerve Brp fluorescence in *srpk79D^atc^* mutants compared to wild-type and heterozygous controls (*p*<0.001, Student *t*-test; Figure 1K). We also found a highly significant increase in the average puncta fluorescence intensity compared to wild-type and heterozygous controls. Indeed, the entire distribution of puncta intensities was shifted toward larger values (*p*<0.001, Mann-Whitney *U* Test; Figure 1L). Finally, we estimate that the frequency of these aberrant accumulations corresponds to 0.03 accumulations per micron of individual motor axon length. From these data, we conclude that Brp-positive puncta in *srpk79D^atc^* mutant axons represent larger, abnormal, protein aggregates compared to observations made in wild-type axons.

**Figure 2:**
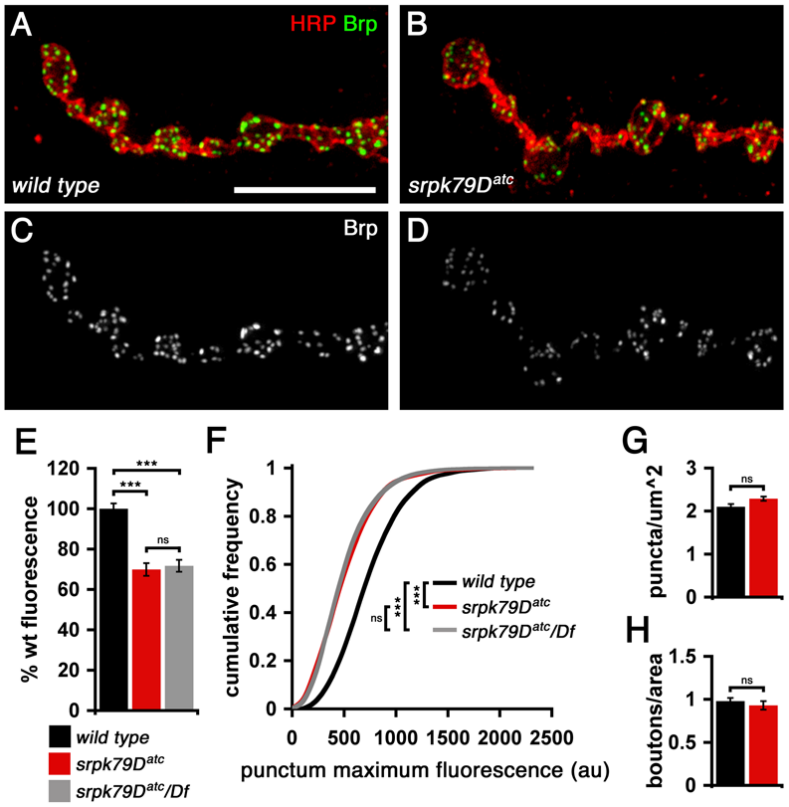
Synaptic Brp deficit in *srpk79D* mutants. (A–D) Immunofluorescence images of wild-type and *srpk79D^atc^* mutant NMJ reveal a synaptic Brp deficit in *srpk79D^atc^* mutants. NMJs are stained with anti-HRP (red) to label the presynaptic membrane and anti-Brp (green). Larvae in wild type and *srpk79D^atc^* were stained in the same reaction tube and imaged identically. (E) The total synaptic Brp fluorescence is decreased in *srpk79D^atc^* mutants. Each bar graph represents data collected from a total of 30 synapses from nine different larvae. (F) Cumulative frequency plots of synaptic Brp puncta fluorescence intensities are shifted toward smaller values with *srpk79D* loss of function. Each curve represents data collected from 30 synapses from nine different larvae. *n* for wild type, *srpk79D^atc^*, and *srpk79D^atc^*/Df = 6,554, 4,713, and 5,952, respectively. au = arbitrary units; HRP = anti-horseradish peroxidase. (G and H) Disruption of *srpk79D* affects neither synaptic Brp puncta number (G) nor synaptic bouton number (H). Each bar graph in (G) and (H) represents data collected from a total of 38 synapses taken from 15 different larvae. Scale bar indicates 10 µm. Significance is indicated according to the following: *** = *p*<0.001 and ns = not significant; Student *t*-test. Error bars indicate ±SEM.

Next, we assayed synaptic Brp staining intensity and NMJ morphology in the *srpk79D^atc^* mutant. We found that synaptic Brp staining intensity is significantly decreased compared to wild-type animals, assayed as both total Brp fluorescence (*p*<0.001, Student *t*-test; Figure 2A–2E) and as the distribution of individual puncta intensities (*p*<0.001, Mann-Whitney *U* Test; Figure 2A–2D and 2F). This effect occurs at NMJ throughout the animal, and there is no evidence for a strong anterior–posterior gradient of this phenotype (Figure S1). Our data suggest that the accumulation of Brp aggregates in the axon of the *srpk79D^atc^* mutant depletes Brp protein from the presynaptic nerve terminal. Consistent with this conclusion, we found that total Brp protein levels, assayed by western blot, are unaltered in the *srpk79D^atc^* mutant background despite the dramatic increase in nerve Brp (see below).

We also determined whether the decrease in total Brp fluorescence causes a decrease in total Brp puncta number, which would be indicative of a change in AZ number. We found, however, that Brp puncta density within *srpk79D^atc^* mutant NMJs is identical to wild type and that total bouton numbers are wild type in the *srpk79D^atc^* mutant background (Figure 2G and 2H). Moreover, anti-Dlg, anti-Synaptotagmin 1 (Syt), and anti-Cysteine String Protein (CSP) staining at *srpk79D^atc^* mutant synapses are not different compared to wild type (unpublished data). Thus, synapse growth, morphology, and AZ number appear normal in the *srpk79D^atc^* mutant.

Consistent with the observed lack of morphological change, we found no change in neurotransmitter release in the *srpk79D^atc^* mutant background. We assayed neurotransmission by recording from the third-instar NMJ of homozygous *sprk79D^atc^* mutants, as well as homozygous *srpk79D^atc^* mutants lacking one copy of the *brp* gene *(brp^69^/+;srpk79D^atc^*) [5]. In all cases, evoked excitatory junctional potential (EJP) amplitude and spontaneous miniature EJP (mEJP) amplitudes were wild type (wild-type average EJP = 34.28±1.59 mV compared to *srpk79D^atc^* = 34.27±1.28 mV; *n* = 10, *p*>0.3; wild type average mEJP = 0.97±0.04 mV compared to *srpk79D^atc^* = 0.99±0.03 mV; *n* = 10, *p*>0.3). There was also no difference in the ability of the NMJ to sustain high-frequency (10 Hz) stimulation in high extracellular calcium saline (2 mM) (unpublished data; see below for additional electrophysiological analyses). Thus, the *srpk79D^atc^* mutant causes inappropriate axonal accumulations of Brp protein, resulting in a depletion of this synaptic protein from the presynaptic AZ. However, the amount of depletion of Brp from the NMJ does not cause a defect in synaptic function over the time course of 4 d of larval development.

### SRPK79D Is Expressed in the Embryonic Central Nervous System and Is Required in Larval Motoneurons

We have used our quantitative assays to confirm that the phenotype of axonal Brp accumulation is caused by disruption of the *srpk79D* gene and to determine the nature of this genetic disruption. First, we demonstrated that the axonal Brp accumulation and synaptic Brp deficit phenotypes in the homozygous *srpk79D^atc^* mutant are statistically identical to those observed when the *srpk79D^atc^* mutation is placed *in trans* to a deficiency chromosome that uncovers the *srpk79D* gene locus, *Df(3L)Exel6138* (Figures 1E–1L, 2E, and 2F). Furthermore, an independently identified molecular null allele of *srpk79D* (*srpk79D^VN100^*; Eric Buchner, personal communication), has axonal and synaptic Brp phenotypes that are statistically identical to those observed in homozygous *sprk79D^atc^* (Figure S2A–S2H). These data are consistent with the conclusion that the *srpk79D^atc^* transposon insertion is a strong loss-of-function or null mutation in the *srpk79D* gene. Interestingly, we found that the heterozygous *srpk79D^atc^/+* mutant axons also have a slight, but statistically significant, increase in Brp fluorescence compared to wild type. These data indicate that *srpk79D* is partially haploinsufficient for the regulation of axonal Brp accumulation.

**Figure 3:**
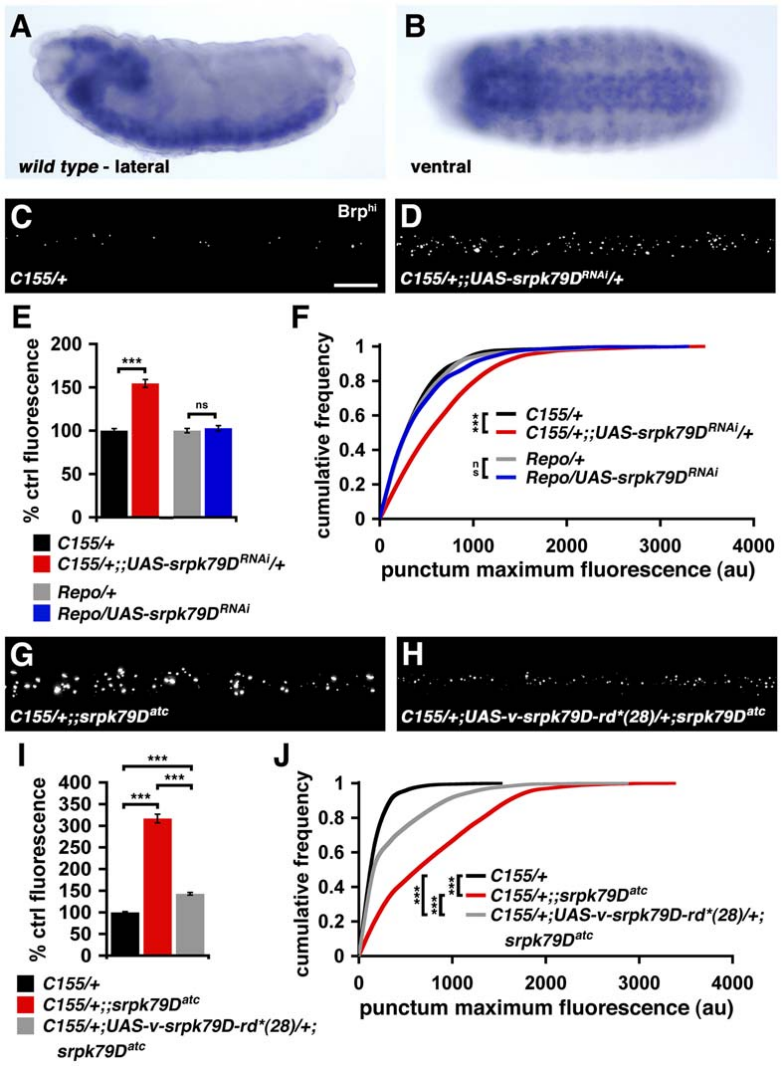
SRPK79D functions in neurons to prevent Brp accumulation. (A and B) Whole-mount in situ hybridizations demonstrate that *srpk79D* is widely expressed but is enriched in the CNS. Anterior is to the left. (C and D) Immunofluorescence images of a control nerve (C) and a nerve expressing *srpk79D*-specific double-stranded RNA (dsRNA) (*UAS*-*srpk79D^RNAi^*) in neurons (D). Expression of dsRNA causes accumulation of Brp puncta. (E and F) Total Brp fluorescence is increased and cumulative frequency plots are shifted toward larger values when *srpk79D^RNAi^* is expressed in neurons, but not when it is expressed in glia using the glia-specific Repo-GAL4 driver. Each bar graph and curve in (E) and (F) represents data collected from a total of 30 nerves from nine different larvae. In (F), *n* for *C155/+*, *C155/+;;UAS-srpk79DRNAi/+*, *Repo/+*, and *Repo*/*UAS*-*srpk79D^RNAi^* = 1,034, 2,451, 893, and 862, respectively. (G–J) Expression of a Venus-tagged *srpk79D* transgene (*UAS*-v-srpk79D-rd*) rescues Brp accumulations in *srpk79D* mutant nerves. (G and H) Nerves are stained with anti-Brp and imaged identically. Brp accumulations are present in the *srpk79D* mutant (G), and these accumulations are less intense following rescue of the *srpk79D* mutant with the *UAS*-*srpk79D* transgene (H). (I and J) Quantification of Brp fluorescence intensity (I) and puncta intensities (J), comparing control (*C155/+*), *srpk79D* mutant (*C155/+;srpk79D^atc^*), and rescue animals (*C155/+*; UAS-v-srpk79D-rd*(28)/+;srpk79D^atc^). Each bar graph and curve in (I) and (J) represent data collected from a total of 36 nerves from nine different larvae. In (J), *n* for *C155/+*, *C155/+;;srpk79D^atc^*, and *C155/+;UAS*-v-srpk79D-rd*(28)/+; *srpk79D^atc^* = 2,452, 2,696, and 3,645, respectively. Scale bar = 10 µm. Significance is indicated according to the following: *** = *p*<0.001 and ns = not significant; Student *t*-test. Error bars indicate ±SEM. au = arbitrary units.

Next, we determined the expression pattern of the *srpk79D* gene. In situ hybridizations performed on wild-type *Drosophila* embryos targeting an exon common to all known *srpk79D* transcripts (see Materials and Methods) detected high levels of *srpk79D* mRNA in the embryonic ventral nerve cord with lower expression present outside of the nervous system (Figure 3A and 3B). This expression pattern is consistent with a function of *srpk79D* gene products in neurons, but does not rule out a possible function in other tissues including peripheral glia.

To confirm that loss of *srpk79D* is responsible for the phenotype of axonal Brp accumulation, and to determine where *srpk79D* is required for normal Brp targeting, we employed a *srpk79D* RNA interference (RNAi) transgene (*UAS-srpk79D^RNAi^*; Vienna *Drosophila* RNAi Collection). We found that expression of *UAS-srpk79D^RNAi^* in neurons phenocopies the *srpk79D^atc^* mutation (Figure 3C–3F), whereas expression of *UAS- srpk79D^RNAi^* in glia (also present in peripheral nerve) does not cause formation of axonal Brp aggregates. These data indicate that *srpk79D* function is required in neurons, consistent with enriched expression in the central nervous system (CNS).

We also performed a genetic rescue experiment by expressing a Venus-tagged, full-length *srpk79D* transgene (UAS-v-srpk79D-rd*) in neurons in the homozygous *srpk79D^atc^* mutant background. In this experiment, neuronal expression of UAS-v- srpk79D-rd* significantly rescued the *srpk79D^atc^* mutant phenotype toward wild-type levels (Figure 3G–3J). The presence of axonal Brp accumulations was reduced (Figure 3G and 3H), and there was a correlated increase in synaptic Brp fluorescence in the rescue animals compared to the mutation (unpublished data). Taken together, our data are consistent with the conclusion that loss of *srpk79D*, in neurons, is responsible for the abnormal accumulation of Brp in peripheral nerves.

Finally, we noted that the *srpk79D* gene resides just downstream of the gene encoding CSP. In mammals, CSP was recently shown to suppress axonal protein aggregation [14]. Therefore, we pursued additional experiments to determine whether disruption of the *Csp* gene might participate in the phenotype of Brp axonal accumulation. In these experiments, we took advantage of a strong hypomorphic *Csp* allele in which the 5′ region of the *Csp* gene is deleted and the *srpk79D* locus is intact (*Csp^X1^*, Figure 1A) [15]. When *srpk79D^atc^* was placed *in trans* to the *Csp^X1^* mutation, we found a modest increase in Brp fluorescence and Brp puncta intensity compared to wild type, but not compared to the *srpk79D^atc^/+* heterozygous mutant (Figure 1K and 1L). On the basis of these data, we conclude that *Csp* is not directly involved in the phenotype of increased axonal Brp puncta staining observed in the *srpk79D* mutant.

### Evidence That Brp Aggregates in *srpk79D* Mutants Are Not Caused by a General Defect in Axonal Transport

**Figure 4:**
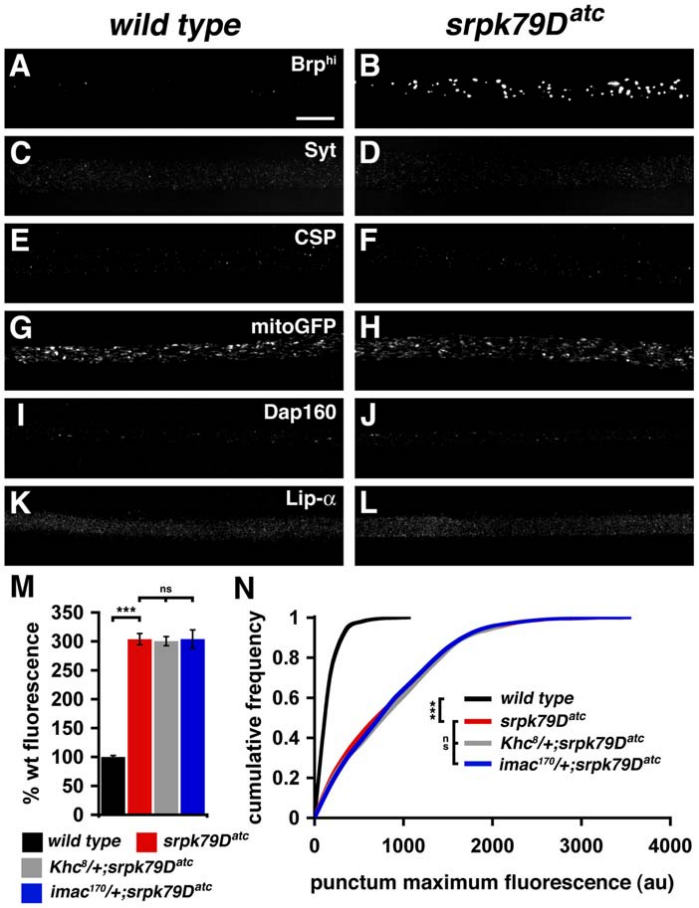
Axonal transport is intact in *srpk79D* mutants. (A–L) Immunofluorescence images of wild-type and *srpk79D* mutant nerves demonstrating the distribution of Bruchpilot (Brp; [A and B]), the synaptic vesicle proteins Synaptotagmin 1 (Syt; [C and D]) and Cysteine String Protein (CSP; [E and F]), mitochondria (mitoGFP; [G and H]), the peri-AZ protein Dap160/Intersectin (Dap160; [I and J]), and the AZ protein Liprin-alpha (Lip-α; [K and L]). Image exposures were selected such that small puncta could be visualized in the wild-type controls, corresponding to a “high exposure” in Figure 1. Wild type and *srpk79D* mutants were stained in the same reaction tube and imaged identically for each antibody. (M and N) Reducing *kinesin heavy chain* (*Khc8/+*) or *immaculate connections* (*imac170/+*) in the *srpk79D* mutant background does not enhance the *srpk79D* mutant phenotype. Each bar graph in (M) and curve in (N) represents data collected from a total of 30 nerves from nine different larvae. In (N), *n* = 1,040, 2,928, 2,560, and 3,240 for wild type, *srpk79Datc*, *Khc8/+*; *srpk79D^atc^*, and *imac170/+*; *srpk79D^atc^*, respectively. Scale bar indicates 10 µm. Significance is indicated according to the following: *** = *p*<0.001 and ns = not significant; Student *t*-test. Error bars indicate ±SEM. au = arbitrary units.

To date, the formation of axonal protein aggregates has been documented in mutations that disrupt both retrograde and anterograde axonal transport [16]–[19]. For example, mutations in *kinesin heavy chain* and disruption of the Dynein/Dynactin protein complex cause large axonal aggregates composed of diverse synaptic proteins and organelles including, but not limited to, Syt, CSP, Dap160/Intersectin (Dap160) mitochondria, and Brp [16],[17],[19]. Thus, we considered the possibility that the *srpk79D^atc^* mutation disrupts axonal transport by asking whether additional synaptic proteins accumulate with Brp in the *srpk79D^atc^* mutant axons. We found, however, that the distribution of Syt, CSP, mitochondria, Dap160, and Liprin-alpha were all unchanged relative to wild type in the *srpk79D^atc^* mutants (Figure 4A–4L). We also find overexpressed EGFP-CaV2.1 is wild type in the *srpk79D^atc^* mutants (unpublished data) [20]. Thus, the *srpk79D^atc^* mutation seems to specifically disrupt the transport or aggregation of the Brp protein in peripheral axons without affecting the transport of synaptic vesicles or other AZ constituent proteins.

We next explored the possibility that SRPK79D participates in the specific transport of Brp protein. In recent years, proteins have been identified that are specifically required for the anterograde transport of synaptic proteins or other cellular organelles such as mitochondria [8],[17],[21]. SRPK79D is not strictly required for axonal transport of Brp because Brp protein is present at the NMJ in the *srpk79D^atc^* mutant. However, it is possible that SRPK79D facilitates the anterograde transport of Brp/T-bars. Therefore, we pursued genetic interactions between *srpk79D* and either *kinesin heavy chain* (*Khc*) or *kinesin 3* (*immaculate connections*; *imac*) [8],[16]. Larval nerves that are heterozygous for the amorphic *Khc^8^* allele contain rare “axonal swellings” that contain Syt, CSP, Brp, Dap160, and KHC [16] (E. L. Johnson and G. W. Davis, unpublished data). Importantly, these swellings can be clearly distinguished from the axonal Brp accumulations observed in *srpk79D* mutants because Brp accumulations in *srpk79D^atc^* do not contain any other known synaptic protein. Therefore, we are able to assess whether the presence of a heterozygous *Khc* or *imac* mutation would enhance the *srpk79D^atc^* mutant phenotype by increasing the abundance of Brp-specific protein aggregates in animals colabeled with anti-Brp and an additional synaptic protein. If SRPK79D facilitates axonal transport of Brp, then reducing KHC or Imac protein levels should enhance the *srpk79D^atc^* mutant phenotype (Brp-specific protein aggregates). However, we found that placing a heterozygous *Khc^8^*/+ or *imac^170^/+* mutation in an *srpk79D^atc^* homozygous mutant background (*Khc^8^/+*; *srpk79D^atc^* or *imac^170^/+*; *srpk79D^atc^*) affected neither the frequency nor the severity of the Brp-specific axon aggregates characteristic of the *srpk79D^atc^* mutant, nor was there any difference in the axonal swellings characteristic of the *Khc* mutant (multiprotein aggregates) (Figure 4M and 4N). We then repeated this experiment using numerous additional mutations in the *Khc* gene as well as other genes implicated in axonal transport including: 1) the antimorphic *Khc^16^* allele [16], 2) *Df(3L)34ex5*, which deletes the *kinesin light chain* locus [22], and 3) the amorphic *dynein heavy chain at 64C* allele, *Dhc64C^4-19^* [23]. We also analyzed double mutants for *srpk79D* and *liprin-alpha (lip-α)*, an AZ protein shown to play a role in axon transport [24] (*lip-α^R60^/lip-α^F3ex5^*; *srpk79D^atc^*). None of these perturbations had any effect on Brp-specific protein accumulations in axons (unpublished data). Finally, through direct observation, we find that small Brp puncta continue to be transported along axons in the *srpk79D^atc^* mutant larval nerves, whereas large aggregates appear to be stalled (Figure S3). Taken together, our genetic and live imaging data support the conclusion that Brp accumulations observed in *srpk79D* mutants are not due to a general defect in axonal transport.

**Figure 5:**
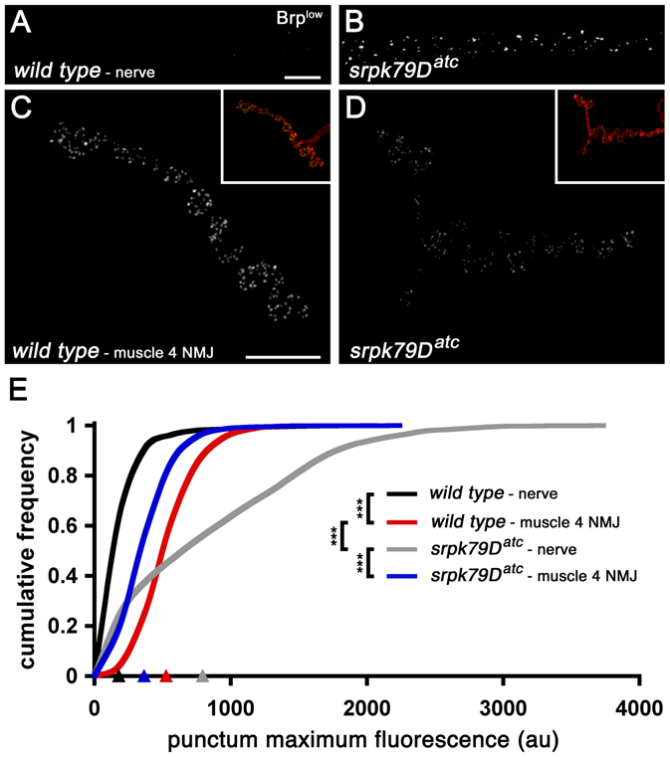
Comparison of synaptic and axonal Brp assemblies in wild-type and *srpk79D* mutant animals. (A–D) Immunofluorescence images of wild-type and *srpk79D* mutant nerves and synapses. Insets in (C) and (D) show the shape of the nerve terminal arborization at lower magnification based upon costaining with anti-Hrp. (E) Cumulative frequency plots of individual Brp puncta fluorescence intensities. Each curve represents data collected from a total of 18 nerves or synapses from nine larvae. Arrowheads on the *x*-axis in (E) indicate the average maximum punctum fluorescence intensity for each genotype (indicated by arrowhead color). Scale bars indicate 10 µm. Significance is indicated according to the following: *** = *p*<0.001; Student *t*-test. Error bars indicate ±SEM.

Finally, we asked whether T-bars might be preassembled structures that are trafficked to the NMJ and inserted at the AZ. In some mutant backgrounds, T-bars have been observed to dislodge from the synapse and reside in the cytoplasm [25]. However, we have never observed the appearance of T-bar–like structures in wild-type *Drosophila* axons at the ultrastructural level (R. D. Fetter and G. W. Davis, unpublished data). This suggests that T-bars are normally assembled at the presynaptic AZ. To examine this question further, we analyzed the size and intensity of anti-Brp puncta in wild-type axons and synapses. At the light level, the vast majority of Brp puncta in wild-type axons are smaller and less intense than the puncta observed within the wild-type presynaptic nerve terminal, suggesting that synaptic T-bars are assembled at the synapse from constituent proteins, including Brp, that are transported down the axon to the synapse (Figure 5). By contrast, Brp puncta observed in *srpk79D* mutants stained more intensely and were much larger than Brp puncta found in wild-type axons. These Brp puncta were also often larger than the T-bar-associated Brp puncta observed at wild-type NMJ (Figure 5). Thus, the large Brp accumulations found in *srpk79D* mutant axons could represent superassemblies of T-bar-related proteins, including Brp. To address this possibility, we examined *srpk79D* mutant axons ultrastructurally.

### Evidence for Premature T-Bar Assembly in *srpk79D* Mutant Axons

Mutations that cause focal accumulation of synaptic proteins in *Drosophila* nerves have been described previously and ultrastructural analyses have been carried out for three of these mutants. In *Khc* and *Dhc64C* mutants, axons become dramatically enlarged and are filled with an array of membrane-bound organelles, including multivesicular bodies, prelysosomal vacuoles, and mitochondria [16],[17]. In contrast, *lip-α* mutant axons have normal diameters and contain organelle accumulations composed predominantly of clear-core vesicles [18].

**Figure 6:**
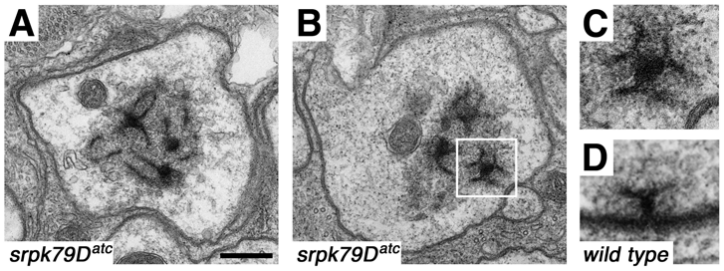
T-bar superassemblies are found in *srpk79D* mutant axons. (A and B) Electron micrographs of *srpk79D* mutant motor axons showing large, electron-dense structures that are never found in wild-type axons. (C) Magnified image of region boxed in (B) highlighting an accumulation that is particularly reminiscent of a superassembly of T-bars. (D) A wild-type synaptic T-bar at the same magnification for comparison to the image in (C). Number of animals sectioned: wild type = 5; mutant = 9. Number of sections analyzed: wild type = 150; *srpk79D^ATC^* = 325. All panels are sized relative to the scale bar shown in (A), as follows: 160 nm for (A and B); 80 nm for (C and D).

In the *srpk79D* mutant, we found that axon diameters were not different from wild type (Figure 6A and 6B). Remarkably, and in contrast to all three of the mutants described above, we found that *srpk79D* mutant motor axons contained highly organized, electron-dense structures that were not surrounded by a vesicular or intracellular membrane compartment (Figure 6A–6C). Often, these electron-dense structures appeared strikingly similar to T-bars that had been joined at their “bases” into a large T-bar aggregate (Figure 6C and 6D). We have never observed a similar structure in wild-type axons. In this study, we performed electron microscopy on five wild-type animals, analyzing 150 sections from the segmental nerves. None of these sections showed evidence of electron-dense aggregates. We performed electron microscopy on nine *srpk79D* mutant animals, analyzing 325 sections from segmental nerves. Sections from every mutant animal showed evidence of electron-dense plaques. Nearly every section from an individual mutant showed evidence of electron-dense plaques, consistent with the highly penetrant phenotype observed at the light level. The dimensions of these electron-dense structures, the prevalence of these structures in our electron microscopy sections and the similarity of their shape to T-bars present at the AZ strongly suggest that these structures represent the large Brp aggregates (superassemblies) that we observe at the light level in the *srpk79D* mutant background. Finally, similar to T-bars found at AZs, these electron-dense structures were surrounded by a filamentous matrix (Figure 6A–6D). Although vesicles were also observed in these areas, we believe that they are molecularly distinct from synaptic vesicles because synaptic vesicle markers do not colocalize with Brp in the *srpk79D* mutant axons (Figure 4A–4L). In contrast, synaptic ultrastructure in *srpk79D* mutants is identical to wild type (unpublished data). Thus, loss of *srpk79D* leads to the formation of T-bar–like superassemblies in axons. Since we have never observed T-bar–like structures in wild-type axons, we propose that SRPK79D is required as part of a mechanism that normally suppresses premature T-bar assembly in the axon.

### SRPK79D Colocalizes with Brp in the Axon and at the Active Zone

To gain insight into the subcellular distribution of SRPK79D, we generated Venus-tagged *srpk79D* transgenes under UAS control (UAS-*v-srpk79D*) and expressed these transgenes in *Drosophila* neurons. We found that neuronally expressed Venus-SRPK79D-RD*, which rescues axonal Brp accumulations (Figure 3G–3J), precisely colocalizes with Brp in both the nerve and at each presynaptic AZ (see below). The voltage-gated calcium channel Cacophony is among very few other proteins that have been demonstrated to colocalize with Brp at the presynaptic AZ [20]. Furthermore, Venus-SRPK79D-RD* is highly unusual in that this protein has been shown to colocalize with Brp in a wild-type axon. These data suggest that SRPK79D closely associates with Brp during axonal transport of the Brp protein to the presynaptic nerve terminal. It is possible that the distribution of the tagged-SRPK79D protein does not reflect the wild-type SRPK79D protein distribution. However, the observation that Venus-SRPK79D-RD* shows a very restricted distribution, colocalizing with Brp in at least two cellular compartments, argues that this protein reflects, at least in part, the normal protein distribution.

**Figure 7:**
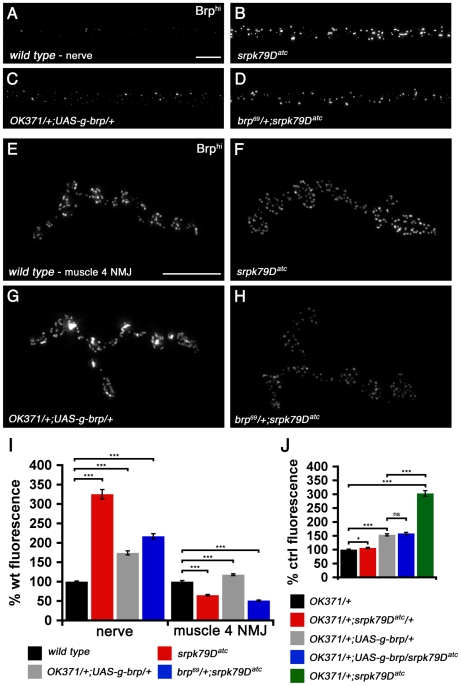
Brp accumulation in *srpk79D* mutants is not due to increased Brp expression. (A and B) Immunofluorescence images of wild-/type and *srpk79D* mutant nerves. (C) Similar Brp accumulations appear when GFP-Brp is overexpressed in motor neurons using the motoneuron-specific GAL4 driver *OK371-GAL4*. (D) Brp accumulations persist in *srpk79D* mutants when one copy of *brp* is deleted by placing a heterozygous *brp^69^/+* mutation in the homozygous *srpk79D* mutant background. (E and F) Immunofluorescence images of wild-type and *srpk79D* mutant muscle 4 NMJ stained with anti-Brp. (G) Synaptic Brp is increased following GFP-Brp overexpression. (H) Removal of one copy of *brp* (*brp^69^*/+) in a homozygous *srpk79D* mutant background causes a further decrease in synaptic Brp levels compared to *srpk79D* mutants alone, but does not cause altered distribution of Brp immunoreactivity. (I) Quantifications of total Brp fluorescence for each indicated genotype normalized to wild type (wt). Each bar graph represents data collected from a total of 29 synapses from 14 different animals. (J) Axonal Brp fluorescence is increased in *srpk79D^atc^/+* heterozygotes (*OK371*/+; *srpk79D^atc^*/+) and when Brp is overexpressed in motor neurons (*OK371*/+; *UAS-g-brp*). An additive effect is seen when these two perturbations are combined (*OK371*/+; *UAS-brp/srpk79D^atc^*). In all cases, total synaptic anti-Brp fluorescence is significantly less than that seen in *srpk79D* mutants (*srpk79D^atc^*). Each bar graph represents data collected from a total of 32 synapses from eight different larvae. Scale bars indicate 10 µm. Significance is indicated according to the following: *** = *p*<0.001 and ns = not significant; Student *t*-test. Error bars indicate ±SEM.

Given that SRPK79D colocalizes with Brp, we considered two hypotheses for SRPK79D function. First, we considered the hypothesis that SRPK79D somehow influences total Brp protein levels in the cell, perhaps by influencing Brp stability or turnover. However, when we assayed total Brp protein levels by western blot, we found no change in the *srpk79D* mutant compared to wild type and no evidence of altered protein degradation (Figure S4B and S4C). Although western blots fail to measure Brp protein levels exclusively in motoneurons, this is consistent with our prior observation that axon Brp fluorescence increases while synaptic Brp decreases in the *srpk79D* mutant, leaving total Brp protein levels constant in the cell. To further examine this possibility, we overexpressed a GFP-tagged *brp* transgene in otherwise wild-type motoneurons using the GAL4-UAS expression system [4]. Although this resulted in the accumulation of Brp protein within axons, GFP-Brp overexpression did not precisely phenocopy the *srpk79D* mutant. GFP-Brp expression simultaneously increased synaptic and axonal fluorescence, whereas the *srpk79D* mutation causes increased axonal Brp and a correlated decrease in synaptic Brp (Figures 1, 2, and 7A–7I). Thus, although Brp overexpression is sufficient to cause axonal aggregates, it seems unlikely that this is the cause of the defect in the *srpk79D* mutant. Consistent with this conclusion, co-overexpression of BRP and SRPK79D-RD* does not reduce the severity of the axonal accumulations caused by BRP overexpression alone. Similarly, axonal accumulations caused by BRP overexpression are not dramatically enhanced by mutating one copy of the *srpk79D* gene (Figure 7J). To further address this issue, we asked whether Brp aggregates form in homozygous *srpk79D^atc^* mutants in which we decrease total Brp levels by removing one copy of the *brp* gene (*brp^69^*/+;*srpk79D^atc^*) [5]. We found that large axonal Brp aggregates persisted even when one copy of the *brp* gene is removed in the background of the *srpk79D^atc^* homozygous mutant (Figure 7D, 7H, and 7I). Taken together, these experiments indicate that an SRPK79D-dependent elevation in Brp protein is not the direct cause of premature T-bar–like assembly formation in the axon. We therefore favor an alternative model based upon the observation that SRPK79D colocalizes with Brp and speculate that SRPK79D could sequester or inhibit the function of axonal T-bar proteins and thereby prevent the formation of axonal T-bar–like superassemblies.

### SRPK79D Kinase Activity Is Necessary to Prevent Brp Accumulation

**Figure 8:**
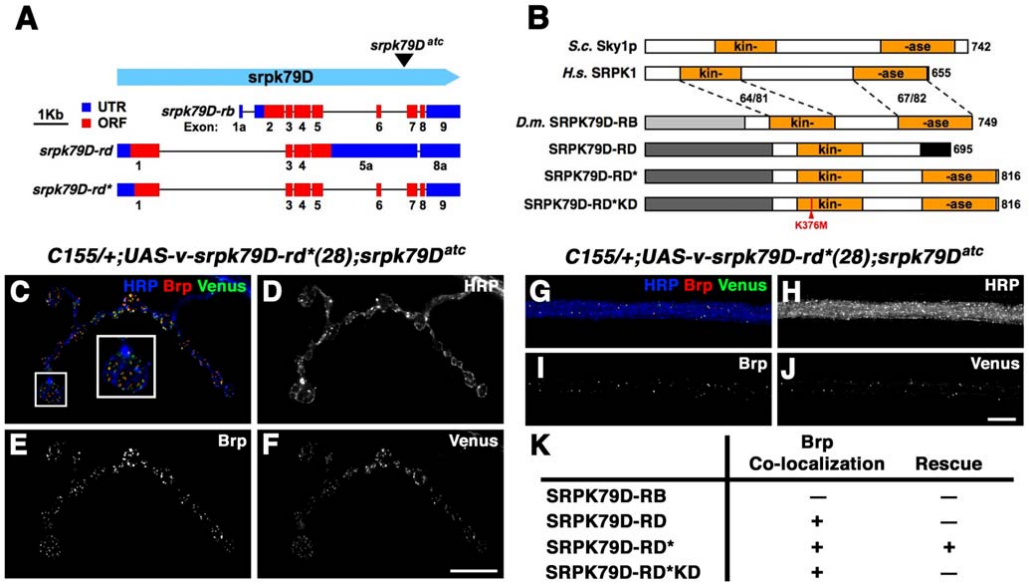
SRPK79D kinase activity is necessary, and the SRPK79D N-terminus directs targeting. (A) A schematic of two previously cloned *srpk79D* transcripts (*srpk79D-rb* and *srpk79D-rd*) 62. *srpk79D-rd* results from read-through of predicted splice sites in exons 5 and 8. We generated the transcript conforming to the predicted splicing pattern of exons 5 and 8 is also shown (srpk79D-rd*). We have since confirmed the existence of this transcript by reverse-transcriptase PCR. (B) A schematic of yeast Sky1p and human SRPK1 protein domain structures as well as domain structures for the predicted srpk79D protein products (SRPK79D-RB, SRPK79D-RD, and SRPK79D-RD*). Alternative exon usage results in a unique SRPK79D-RB N-terminus (light gray), whereas SRPK79D-RD and SRPK79D-RD* use the same N-terminus (dark gray). The splicing pattern employed in SRPK79D-RD leads to a truncated kinase domain relative to SRPK79D-RB* and SRPK79D-RD. SRPK79D-RD*KD contains a missense mutation at position 376 (K376M) that targets the predicted ATP binding site. Numbers indicate the percent amino acid identity/similarity of the SRPK79D kinase domain to that of human SRPK1. *D.m.* = *Drosophila melanogaster*; HRP = anti-horseradish peroxidase; *H.s.* = *Homo sapiens*; *S.c.* = *Saccharomyces cerevisiae*. (C–F and I–J) Immunofluorescence images of synapses and nerves, respectively, demonstrate near-perfect colocalization between Brp and Venus-SRPK79D-RD* in both the NMJ (C–F) and in the axons (G–J). (K) Table indicating Brp colocalization and ability to rescue axon Brp accumulation phenotype of the *srpk79D* gene products indicated in (A) and (B). Scale bars indicate 10 µm. Error bars indicate ±SEM. See also Supplemental Discussion (Text S1) relevant to this annotation.

As mentioned above, sequence analysis of the predicted *srpk79D* gene products reveals similarity to a group of serine-threonine kinases called SRPKs (Figure 8B). Members of this protein kinase family share a characteristic split serine-threonine kinase domain [26]. We therefore performed experiments to determine whether the kinase domain is required for SRPK79D activity. Transgenically expressed full-length SRPK79D (SRPK79D-RD*) colocalizes with Brp and rescues the *srpk79D* mutant phenotype (Figure 8C–8K). In contrast, expression of an SRPK79D isoform with a truncated kinase domain (SRPK79D-RD) colocalized with Brp, but failed to rescue the *srpk79D* mutant phenotype (Figure 8B and 8K). These data indicate that the SRPK79D kinase domain is involved in preventing axonal superassemblies of Brp but that it is not important for colocalization with Brp. To further test the importance of SRPK79D kinase activity, we generated a kinase dead *srpk79D* transgene by introducing a missense mutation into SRPK79D-RD* that is predicted to disrupt the ATP binding pocket of the kinase domain and thereby inhibit kinase activity (SRPK79D-RD*KD, Figure 8B). A similar strategy has been used previously to eliminate kinase activity in other SRPKs ranging from yeast to human [27]–[29]. Like SRPK79D-RD*, SRPK79D-RD*KD colocalized with Brp. However, even when expressed at higher levels than SRPK79D-RD*, SRPK79D-RD*KD failed to rescue the *srpk79D* mutant phenotype (Figure 8K and unpublished data).

We next asked which domains might be required for SRPK79D protein trafficking and/or localization. We have found that an SRPK79D transgene that possesses an alternative SRPK79D N-terminal region but is otherwise identical to SRPK79D-RD* (SRPK79D-RB) failed to be efficiently trafficked out of the neuronal soma, was not found to colocalize with Brp, and failed to rescue the *srpk79D* mutant phenotype (Figure 8B and 8K and unpublished data). This suggests that the common N-terminal domain of SRPK79D-RD, SRPK79D-RD*, and SRPK79D-RD*KD is required for the axonal transport of SRPK79D and its colocalization with Brp.

### Overexpression of SRPK79D Disrupts Synaptic Brp and Impairs Synaptic Function

**Figure 9:**
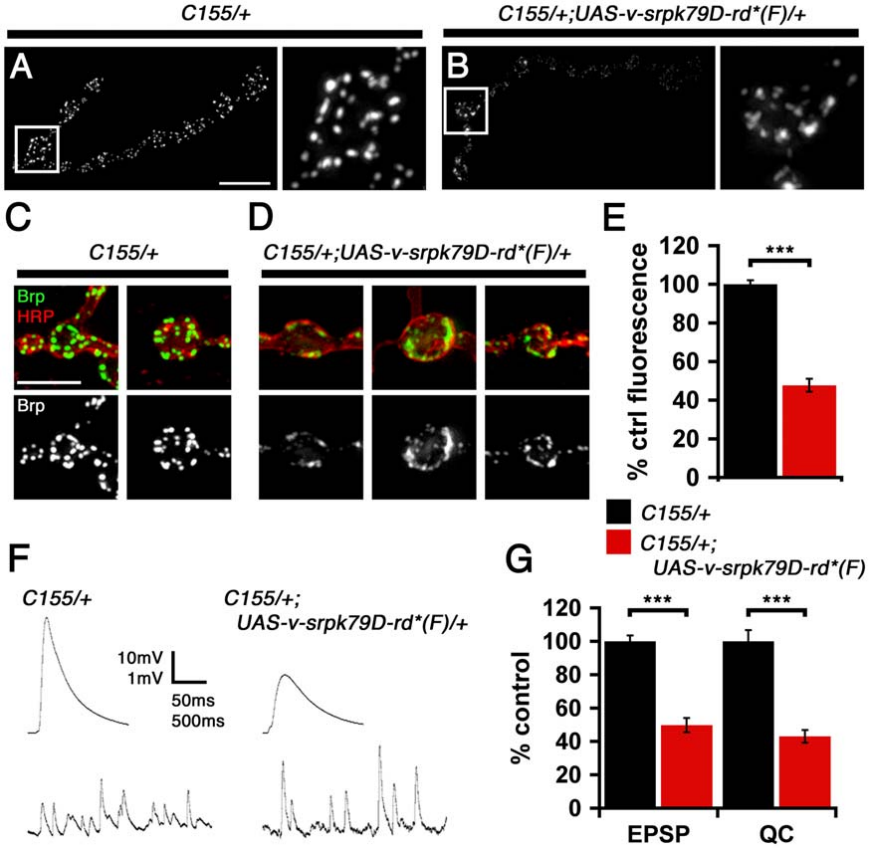
SRPK79D overexpression disrupts synaptic Brp and impairs synapse function. (A–D) Representative muscle 4 NMJ and individual bouton from control (*C155*/+) and SRPK79D-RD*-overexpressing larvae demonstrating diffuse Brp staining and reduced total Brp fluorescence. Image offset and gain in (A) and (B) are identical. (C) Example of type-1b boutons from control animals demonstrating typical punctate anti-Brp staining (green) and anti-HRP staining (red) to elucidate the nerve terminal membrane. (D) Examples of diffuse Brp staining observed at type-1b boutons within the NMJ of an SRPK79D overexpressing animal. Image offset and gain in (C) and (D) are identical. (E) SRPK79D-RD* overexpression causes a decrease in total synaptic Brp fluorescence. (F) Overexpression of SRPK79D-RD* causes a highly significant decrease in EPSP amplitude. There is a trend toward an increase in the average amplitudes of spontaneous miniature events comparing SRPK79D-RD*-overexpressing animals to wild type (*p* = 0.06); representative mEPSPs are shown. (G) Quantification of average EPSP amplitude and quantal content (QC) in SRPK79D-RD*-overexpressing larvae show a greater than 50% decreases in both measures relative to control. *C155*/+ and *C155*/+;UAS-v-srpk79D-rd*(F)/+ bar graphs represent data collected from a total of 15 synapses from six different larvae and 12 synapses from five different larvae, respectively. Scale bar in (A) indicates 10 µm, and in (C) indicates 5 µm. Significance is indicated according to the following: *** = *p*<0.001; Student *t*-test. Error bars indicate ±SEM. HR = anti-horseradish peroxidase.

Our data are consistent with a model in which SRPK79D prevents premature assembly of T-bars within axons. This model also suggests that SRPK79D activity must be inhibited locally, at the AZ, in order for synaptic T-bar assembly to proceed. We reasoned that overexpressing SRPK79D might overwhelm the synaptic machinery that disrupts SRPK79D activity and thereby reveal a role for SRPK79D during T-bar assembly or synaptic function. Here, we show that SRPK79D overexpression disrupts the punctate, highly organized appearance of synaptic Brp immunoreactivity (Figures 9A–9D and S5A–S5D). For example, we observed regions where Brp was diffusely organized near the synaptic membrane. These regions encompass areas that would normally contain several individual Brp puncta. We hypothesize that these regions of diffuse Brp reflect either failed T-bar assembly or severely perturbed AZ organization. In addition, we found that SRPK79D overexpression also led to a decrease in total synaptic Brp fluorescence (Figure 9E). This might be consistent with perturbed AZ formation but is also similar to that found in homozygous *srpk79D* mutants (*srpk79D^atc^*), mutants heterozygous for a null mutation in *brp* (*brp^69^/+*), and mutants heterozygous for the brp null mutation and homozygous for the *srpk79D^atc^* allele (*brp^69^/+*; *srpk79D^atc^*; Figures 2 and 7, and unpublished data). It should be noted, however, that the diffuse synaptic Brp staining caused by SRPK79D overexpression is not observed in any of these *srpk79D* or *brp* loss-of-function paradigms. It should be further noted that SRPK79D levels in this overexpression experiment are higher than the SRPK79D levels that are sufficient to rescue the *srpk79D* mutation (Figures 3G–3J, 9, and S5). SRPK localization was determined in rescue animals expressing relatively low levels of transgene-derived SRPK79D, and we believe that this is why we observe normal synaptic architecture and SRPK79D localization in those experiments (Figure 8C–8F). Finally, overexpression of SRPK79D-RD*KD (kinase dead) or SRPK79D-RD (truncated kinase domain) did not cause diffuse Brp staining (unpublished data), indicating that the kinase domain is required for this phenotype.

If SRPK overexpression perturbs T-bar assembly or organization, then we might expect a disruption of presynaptic vesicle release. When we assayed synaptic function in larvae overexpressing SRPK79D, we found a dramatic (∼50%) decrease in excitatory postsynaptic potential (EPSP) amplitude along with a trend toward an increase in the average amplitude of spontaneous miniature events (minis, Figure 9F and 9G). Estimating the average number of vesicles released per action potential (quantal content; calculated according to the average EPSP/average mEPSP per NMJ), we found that quantal content was severely perturbed. Since synapse function is intact in *srpk79D^atc^* homozygous animals, *brp^69^/+* heterozygous animals, and *brp^69^/+*; *srpk79D^atc^* double-mutant larvae (see above), the defects caused by SRPK79D overexpression are likely a consequence of excess SRPK79D activity at AZs. In addition, overexpression of SRPK79D-RD*KD (kinase dead) or SRPK79D-RD (truncated kinase domain) did not cause a defect in synaptic function (unpublished data) indicating that the kinase domain is required for this overexpression phenotype. Finally, it is worth noting that the defects in synaptic function caused by SRPK79D-RD* overexpression are similar to those found in *brp* null mutants, which lack T-bars [5].

## Discussion

Here, we present the identification and characterization of a novel serine-threonine kinase termed Serine-Arginine Protein Kinase at 79D (SRPK79D) that colocalizes with the T-bar-associated protein Brp in both the axon and at the mature AZ. SRPK79D is one of very few proteins known to localize to T-bars or ribbon-like structures at the AZ and is the only known kinase to localize to this site [1]–[5] (Figure 8). We further provide genetic evidence that SRPK79D functions to represses the premature assembly of T-bars in axons. In particular, we show that loss-of-function mutations in *srpk79D* cause the appearance of T-bar–like protein aggregates throughout peripheral axons, and we are able to rule out the possibility that this is an indirect consequence of impaired axonal transport (Figures 4 and 6). The appearance of ectopic T-bars is highly specific since numerous other synaptic proteins and mitochondria are normally distributed in the neuron and are normally trafficked to the presynaptic nerve terminal in the *srpk79D* mutant background (Figure 4A–4L). Thus, SRPK79D appears to have a specific function in repressing T-bar assembly prior to the AZ, consistent with the strong colocalization of SRPK79D protein with Brp and T-bar structures.

Finally, we also uncover a potential function for SRPK79D at the AZ where it is observed to colocalize with Brp. SRPK79D loss-of-function mutations do not alter the number, density, or organization of Brp puncta at the synapse and do not alter synaptic function (Figure 2 and unpublished data). This is consistent with a negative regulatory role for SRPK79D during T-bar assembly and indicates that once SRPK79D-dependent repression of T-bar assembly is relieved, AZ assembly proceeds normally. Overexpression of SRPK79D, however, severely disrupts neurotransmission. The defect in presynaptic release is correlated with a disruption of Brp puncta organization and integrity. These phenotypes are consistent with a function for SRPK79D as a negative regulator of T-bar assembly and AZ maturation.

SRPK79D is a member of the SRPK family of constitutively active cytoplasmic serine-threonine kinases that target serine-arginine–rich domains of SR proteins [26]–[29]. Thus, it is interesting to postulate what the relevant kinase target might be. Given that SRPK79D and Brp colocalize, an obvious candidate is the Brp protein itself. However, the Brp protein does not have a consensus SR domain, and decreasing the genetic dosage of *srpk79D* does not potentiate axonal Brp accumulations that appear upon Brp overexpression [4] (Figure 7J). As such, Brp may not be the direct target of SRPK79D kinase activity. We hypothesize, therefore, that SRPK79D colocalizes with Brp and another putative SR protein that is the direct target of SRPK79D kinase activity.

### Potential Models for SRPK79D-Dependent Negative Regulation of T-Bar Assembly

The best-characterized role for SRPKs is in controlling the subcellular localization of SR proteins, thereby regulating their nuclear pre-mRNA splicing activity [12]. More recently, SR protein involvement in several cytoplasmic mRNA regulatory roles has been reported [30],[31]. In particular, a phosphorylation-dependent role for SR proteins has been reported in both *Drosophila* and mammalian cell culture [32],[33].

It is interesting to speculate that the function of SRPK79D to prevent premature T-bar assembly might be related to the established function of SRPKs and SR-domain-containing proteins during RNA binding, processing, and translation [12],[30]. One interesting possibility is that RNA species are resident at the T-bar. In such a scenario, SRPK79D-dependent repression of RNA translation could prevent T-bar assembly in the axon, and relief of this repression would enable T-bar assembly at the AZ. The continued association of SRPK79D with the AZ could allow regulated control of further T-bar assembly during development, aging, and possibly as a mechanism of long-term synaptic plasticity. Several results provide evidence in support of such a possibility. First, local translation has been proposed to control local protein concentration within a navigating growth cone [34],[35]. There is also increasing evidence in support of local translation in dendrites and for the presence of Golgi outposts that could support local protein maturation [36],[37]. A specific role for RNA binding proteins at the presynaptic AZ is supported by the prior identification of the RIBEYE protein, which is a constituent of the vertebrate ribbon structure. RIBEYE contains a CtBP domain previously shown to bind RNA [2]. The discovery of a different RNA binding protein (CtBP1) at the ribbon and our description of a putative RNA regulatory protein at the *Drosophila* T-bar further suggest that RNA processing might be involved in the formation or function of these presynaptic electron dense structures [3].

In light of these data, we explored the possibility that SRPK79D might participate in translational control related to T-bar assembly. We, therefore, examined mutations in genes that could represent SRPK79D-dependent negative regulators of translation, such as *aret* (*bru*), *cup*, *pum*, *nos*, and *sqd* [38]–[44], reasoning that the loss of such a translational inhibitor might result in the ectopic synthesis of AZ proteins, ultimately leading to a phenotype similar to that observed in *srpk79D* mutants. We also generated genomic deletions for *bru2* and *bru3*. However, we did not find evidence of axonal Brp aggregation in any of these mutants. Next, we assayed mutations previously shown to be required for mRNA transport and local protein synthesis. If necessary for T-bar assembly, these mutations might disrupt synaptic Brp-dependent T-bar formation. These mutations, including *orb*, *vas*, and *stau*, have phenotypes at earlier stages of development, but show no defect in synaptic Brp staining [38],[45]–[48]. Thus, although these experiments do not rule out a function for SRPK79D in local translation, we have examined mutations in several additional candidates and failed to uncover evidence in support of this model.

Another possibility is that SRPK79D inhibits T-bar assembly through the constitutive phosphorylation-dependent control of a putative SR protein that colocalizes with SRPK79D and Brp within a nascent T-bar protein complex. Upon arrival of this nascent T-bar protein complex at the presynaptic nerve terminal, T-bar assembly could be initiated in a site-specific manner through the action of a phosphatase that is concentrated at a newly forming synapse. There are several examples of phosphatases that can be localized to sites of intercellular adhesion, some of which have been implicated in the mechanisms of synapse formation and remodeling [49]. This model, therefore, proposes that negative regulation of T-bar assembly, via SRPK79D, is a critical process required for the rapid and site-specific assembly of the presynaptic AZ-associated T-bar structure. Finally, we can not rule out the possibility that SRPK79D normally functions to prevent T-bar superassembly as opposed to T-bar assembly per se. Consistent with this idea is the observation of T-bar aggregates in axons and prior observation that detached ribbon structures coalesce into large assemblies in vertebrate neurons [50].

### Controlling the Process of Synapse Assembly through Negative Regulation

Synapse assembly is a remarkably rapid event. There is evidence that the initial stages of synapse assembly can occur in minutes to hours, followed by a more protracted period of synapse maturation [11],[51]–[53]. Synapses are also assembled at specific sites. In motoneurons and some central neurons, synapses are assembled when the growth cone reaches its muscle or neuron target [53],[54]. However, many central neurons form en passant synapses that are rapidly assembled at sites within the growing axon, behind the advancing growth cone [53],[54]. Current evidence supports the conclusion that intercellular signaling events mediated by cell adhesion and transmembrane signaling specify the position of the nascent synapse [54]–[56]. The subsequent steps of presynaptic AZ assembly remain less clear. Calcium channels and other transmembrane and membrane-associated proteins appear to be delivered to the nascent synaptic site via transport vesicles that fuse at the site of synapse assembly [9]–[11]. It has been proposed that cytoplasmic scaffolding molecules then gradually assemble at the nascent synapse by linking to the proteins that have been deposited previously [11]. This model assumes, however, that the protein–protein interactions between the numerous scaffolding molecules that comprise the presynaptic particle web do not randomly or spontaneously occur in the cytoplasm prior to synapse assembly. What prevents these scaffolds from spontaneously assembling in the small volume of an axon, prior to synapse formation at the nerve terminal and between individual en passant synapses? Currently, nothing is known about how premature scaffold assembly is prevented. We propose that our studies of *srpk79D* identify one such mechanism of negative regulation that prevents premature, inappropriate assembly of a presynaptic protein complex. We further propose that such a mechanism of negative regulation, when relieved at a site of synapse assembly, could contribute to the speed with which presynaptic specializations are observed to assemble.

## Materials and Methods

### Fly Stocks

The listed strains were obtained from the following sources: *srpk79D[atc]* (*c00270*), *f00171*, *d09582*, *f05463*, and *d09837* from the Exelixis collection at Harvard Medical School; v*47544* (*UAS-srpk79D^RNAi^*) from the Vienna Drosophila RNAi Collection; *P{GawB}elav^C155^* (*C155*), *P{GawB}sca^109-68^* (*Sca*), *P{GawB}OK371 (OK371)*, *P{GAL4}repo* (*Repo*), *Khc^8^*, *Khc^16^*, *Df(3L)34ex5*; *dhc64C^4-19^*, *Df(3L)Exel6138*, *UAS-mitoGFP*, *cup^1^*, *sqd^j4b4^*, *pum^13^*, *nos^L7^*, *vas^RJ36^*, *orb^dec^*, *stau^1^*, and *stau^ry9^* from the Bloomington Stock Center; *Csp^X1^* was a generous gift from Konrad Zinsmaier; *srpk79D^VN100^* was a generous gift from Erich Buchner; *imac^170^* was a generous gift from Thomas Schwarz; *aret^PA^*, *aret^PD^*, and *aret^QB^* were generous gifts from Paul MacDonald; and *UAS-gfp-brp* (*UAS-g-brp*) and *brp^69^* were generous gifts from Stephan Sigrist.

### Immunohistochemistry

Wandering third-instar larvae were dissected in calcium-free saline and fixed with either 4% paraformaldehyde/PBS (15 min) or 100% Bouin's Solution (2 min). Excess fixative was removed by extensive washing in PBS+0.1% Triton-X (PBT). Dissected larvae were then incubated overnight at 4°C in PBT with one or more primary antibodies, washed in PBT, incubated either overnight (4°C) or for 1 h (22°C) in PBT with one or more fluorescent-conjugated secondary antibodies, and washed again before being mounted on a slide for imaging analysis. Primary antibodies: NC82 (anti-Brp; Developmental Studies Hybridoma Bank) 1∶100; 3H2 2D7 (anti-Syt; Developmental Studies Hybridoma Bank) 1∶25; anti-Liprin-alpha (a generous gift from David Van Vactor) 1∶1,000; 1G12 (anti-DCSP-3; Developmental Studies Hybridoma Bank) 1∶25; and anti-Dap160 (Marie et al., 2004 [57]) 1∶100. Fluorescent-conjugated secondary antibodies: goat-anti-mouse Alexa 488 (Invitrogen) 1∶500; goat-anti-mouse Alexa 555 (Invitrogen) 1∶500; and goat-anti-rabbit Alexa 488 (Invitrogen) 1∶500. Where applicable, anti-HRP-Cy3 (Jackson Immunoresearch) 1∶200; anti-HRP-FITC 1∶100 or anti-HRP-Cy5 1∶50 were used at the same step as secondary antibody incubation. Genotypes being directly compared were grouped together during all of the above procedures.

### Imaging and Analysis

Images were digitally captured using a cooled CoolSnapHQ CCD camera mounted on a Zeiss Axiovert 200 M microscope. Images were acquired and analyzed using Slidebook software (Intelligent Imaging Innovations). Individual nerves/synapses were optically sectioned at 0.5 µm (11–27 sections per nerve) using a piezoelectric-driven *z*-drive controlling the position of a Zeiss 100× oil immersion objective (numerical aperture [NA] = 1.4). The intensity of anti-BRP immunostaining was quantified as follows: Each series of 0.5-µm optical nerve sections was deconvolved (nearest-neighbors; Intelligent Imaging Innovations). Two-dimensional projections of the maximum pixel intensity were then generated, and the total Brp fluorescence and the maximum fluorescence intensity of each Brp punctum within the nerve/synapse area were determined for each resulting image using a semiautomated procedure as described previously [58],[59]. For all quantifications, the nerve/synapse area was defined as that delimited by anti-HRP staining.

### Live Imaging

Live imaging was carried out as previously described [60]. In brief, wandering third-instar larvae were dissected in HL3 saline (0.4 mM Ca^2+^) on a glass coverslip and held in place using pressure pins. Images were digitally captured using a Photometrics Cascade 512B camera mounted on an upright Zeiss Axioskop 2 microscope using a 100× water immersion (NA = 1.0) objective and a GFP filter set (Chroma). Time-lapse images were collected and analyzed using Slidebook software (Intelligent Imaging Innovations).

### Whole-Mount mRNA In Situ Hybridization

*srpk79D* mRNA was detected using a protocol based upon the “96-well plate RNA in situ protocol” available at the Berkeley *Drosophila* Genome Project (BDGP) Web site (http://www.fruitfly.org). In short, mixed-stage embryos were collected, fixed in 3.7% formaldehyde/1×PBS, and prepared for incubation with SP6 or T7 polymerase generated digoxigenin (DIG)-labeled nucleotide probes. To generate probes, a 954-base pair (bp) fragment of the *srpk79D* gene was amplified by PCR from cDNA AT02150, obtained from the Berkeley *Drosophila* Genome Project using primers with the sequence 5′-ttacccggattcgtccgac-3′ and 5′-gcagtgattttcttctccgttcgg-3′. This fragment was TA cloned into the pGEM-T Easy vector (Promega). The resulting product was used as a template for T7/SP6 DIG-labeled RNA probe synthesis (Roche). After incubation and removal of excess probe, embryos were incubated with alkaline-phosphatase-conjugated anti-DIG Fab fragments (Roche). Excess Fab fragments were removed by washing, and a NBT/BCIP developing reaction was performed (Roche).

### Northern Blots

Adult heads were removed by freezing at −70°C, followed by agitation. Heads were isolated using mesh filters. RNA was extracted using TRIzol reagent and standard molecular biology techniques. DIG-labeled RNA probes were generated by amplifying an 800-bp fragment of the *brp* gene from cDNA IP09541 obtained from the Berkeley *Drosophila* Genome Project using primers with the sequence 5′-gcaatgggcagtccatactacc-3′ and 5′-cccattcccttggcctgc-3′ and the 738-bp insert from rp49 cDNA RE59709 obtained from the Berkeley *Drosophila* Genome Project and 5′-cggcaaggtatgtgcg-3′ and 5′-actaaaagtccggtatattaacgtttac-3′ and TA cloning into pGEM-T Easy (Promega). The resulting product was used as a template for T7/SP6 DIG-labeled RNA probe synthesis (Roche). Northern blot analysis was carried out using Ambion NorthernMax-Gly protocols and reagents. Probe detection was carried out using alkaline phosphatase-conjugated anti-DIG Fab fragments (Roche) in conjunction with the DIG Wash and Block Kit and CSPD Ready-to-Use.

### Western Blots

Third-instar larval brains were pulverized in 2× Laemmli sample buffer. Proteins were separated by SDS-PAGE and transferred to PVDU membrane. The membrane was blocked in 2% milk powder in 1×TBS-Tween, and then incubated for 1 h at room temperature with an anti-Brp monoclonal antibody (Developmental Studies Hybridoma Bank NC82, 1∶100) or anti-GFP monoclonal antibody (Invitrogen 3E6, 1∶100). As a protein loading control, the membrane was co-incubated with an anti-β-tubulin monoclonal antibody (Developmental Studies Hybridoma Bank E7, 1∶1,000). After washing in 1×TBS-Tween, the membrane was incubated for 1 h at room temperature with horseradish peroxidase-conjugated anti-mouse secondary antibody (1∶20,000), washed again and an electrogenerated chemiluminescence (ECL) detection reaction (Amersham) was performed.

### Electron Microscopy

Mutant and wild-type third-instar larvae were prepared for electron microscopy as follows. Larvae were filleted in physiological saline and fixed with 2% glutaraldehyde in 0.12 M Na-cacodylate buffer (pH 7.4, 10 min). The fixed larvae were then transferred to vials containing fresh fixative and fixed for a total of 2 h with rotation. Larvae were rinsed with 0.12 M Na-cacodylate buffer and postfixed with 1% osmium tetroxide in 0.12 M Na-cacodylate buffer for 1 h. Specimens were then rinsed with 0.12 M Na-cacodylate buffer, followed by water, and then stained en bloc with 1% aqueous uranyl acetate for 1 h. After water rinse, dehydration, and embedding in Eponate 12 resin, sections were cut with a Leica Ultracut E microtome, collected on Pioloform-coated slot grids, and stained with uranyl acetate and Sato's lead. Sections were photographed with a Tecnai spirit operated at 120 kV equipped with a Gatan 4 k×4 k camera.

### Electrophysiology

Recordings were taken in HL3 saline (Ca^2+^ 0.4 mM, Mg^2+^ 10 mM) from muscle 6 in abdominal segments 2 and 3 of third-instar larvae as previously described [61]. Only recordings with resting membrane potentials more negative than −60 mV and input resistances greater then 7 MΩ were used for analysis. Measurements of EPSP and spontaneous miniature release event amplitudes were made using MiniAnalysis software (Synapsoft).

## Supporting Information

Figure S1 **Analysis of synaptic Brp levels in anterior and posterior segments from wild type and** ***srpk79D^atc^*** **mutants.** Cumulative frequency plots of individual muscle 4 NMJ Brp puncta maximum pixel intensities from wild-type abdominal segment A3 (black curve), *srpk79D^atc^* segment A3 (red curve), wild-type segment A6 (grey curve), and *srpk79D^atc^* segment A6 (blue curve). There is a slight, though statistically significant, increase in synaptic Brp puncta intensity in wild-type segment A6 (posterior) compared to A3 (anterior). In homozygous *srpk79D^atc^* mutants, there is a smaller difference comparing anterior and posterior segments. In conclusion, there is a large decrease in Brp synaptic intensity in the mutant compared to wild type, and this is not dramatically affected by the position of the NMJ along the anterior-posterior axis. Each curve represents data collected from synaptic arbors of a total of 36 synapses from 18 larvae. *n* = 4,657, 3,518, 6,134, and 4,638, respectively. * = p<0.05, ** = p<0.01, *** = p<0.001, Mann-Whitney *U* Test.
(0.14 MB TIF)

Figure S2 **Analysis of an additional srpk79D mutation.** (A–C) Representative immunofluorescence images of individual larval nerves stained with anti-Brp antibody from wild-type (A), *srpk79D^atc^* (B), and *srpk79D^VN100^* (C) larvae. (D–F) Representative immunofluorescence images of muscle 4 NMJs stained with anti-Brp antibody from wild-type (D), *srpk79D^atc^* (E), and *srpk79D^VN100^* (F) larvae. (G and H) Bar graphs showing average (±the standard error of the mean [SEM]) nerve (G) and muscle NMJ (H) staining intensities. The increases in nerve staining and decreases in synaptic NMJ in the two *srpk79D* mutant conditions are both highly statistically significant compared to wild type and statistically indistinguishable from each other. Each bar represents data taken from 24 synapses from 12 larvae. *** = p<0.001, Student *t*-test. Scale bars indicate 10 µm.
(0.32 MB TIF)

Figure S3 **Live image analysis.** (A) Live GFP fluorescence image of nerves from a *srpk79D^atc^* mutant larvae expressing GFP-tagged Brp in motoneurons at time = 0 s. Several small and large GFP-Brp puncta are apparent. Scale bar indicates 5 μm. (B–E) show a time series (*t* = 0 to *t* = 6 s) of upper-left boxed region in (A) demonstrating a motile small BRP punctum and an immobile large BRP aggregate. In (B–E), white arrows indicate the GFP-Brp punctum/aggregate position at the beginning of the time series. Red arrows highlight the new position of the motile punctum in each frame. In these experiments, we sought to directly assess Brp transport in *srpk79D^atc^* mutants through live imaging of *srpk79D^atc^* mutant larval nerves expressing a GFP-tagged brp cDNA in motoneurons [4] (*OK371*/+;*UAS-g-brp*, *srpk79D^atc^*/*srpk79D^atc^*). A similar method was used previously to observe GFP-tagged synaptotagmin transport [18]. In our experiments, a variety of GFP-Brp species were observed including small puncta and large accumulations. The majority of small GFP-Brp puncta were found to be motile with individual puncta exhibiting anterograde and/or retrograde transport during a given imaging epoch. The average rate of small GFP-Brp puncta transport was 0.08±0.009 µm/s (average±SEM). Although this rate is somewhat slower than values previously reported for the transport of GFP-tagged Syt, it falls within the range of reported values [18]. In contrast, large GFP-Brp accumulations were generally immotile (as shown here). Occasionally, a large accumulation was observed to alternately exhibit anterograde and then retrograde transport. However, total displacement was never more than 0.3 µm (unpublished data).
(0.43 MB TIF)

Figure S4 **Supplemental molecular analyses.** (A) Northern blot analysis of Brp transcripts in mRNA purified from wild-type (left lane) or homozygous *srpk79D^atc^* mutant (right lane) adult fly heads indicates no difference in mRNA processing between the two genotypes. Approximately 10 µg of mRNA was loaded in each lane. An RNA sequence complementary to a region of the Brp mRNA found in all known transcripts was used to probe the blot. The lower panel shows rp49 mRNA signal as a loading control. (B) Example western blot of protein extracted from wild-type (left lane) or homozygous *srpk79D^atc^* mutant (right lane) larval CNSs. There is no detectable change in Brp expression levels. Bar graph showing average (±SEM) wild-type and *srpk79D^atc^* anti-Brp band intensities (normalized to anti-β-tubulin band intensity) shows that Brp is not overexpressed as a consequence of *srpk79D* loss of function. Bars represent data taken from six independent experiments.
(0.51 MB TIF)

Figure S5 Overexpression of SRPK79D-RD* causes synaptic Brp disruption. (A) Example anti-Brp immunofluorescence image of a muscle 4 NMJ from an animal expressing high levels of SRPK79D-RD*. Inset shows the same synapse including both anti-Brp immunofluorescence (green) and anti-HRP immunofluorescence (red) channels for orientation. Arrowheads highlight boutons with disrupted anti-Brp staining, which are magnified in (B–D). (B–D) Magnified images of highlighted boutons in (A). (E) Western blot analysis of protein extracted from three independent UAS-v-srpk79D-rd* transgenic lines showing differential protein expression. (F) Table summarizing the ability of each transgenic line in (E) to rescue the *srpk79D* mutant phenotypes and to disrupt Brp organization. Scale bar indicates 10 µm.
(0.74 MB TIF)

Text S1 **Supplemental discussion relevant to** **Figure 8** **.**
(0.03 MB DOC)

## Abbreviations

AZ: active zone
CNS: central nervous system
EPSP: excitatory postsynaptic potential
NMJ: neuromuscular junction
RNAi: RNA interference
