# Supplementary figures and images for: Negative Regulation of Active Zone Assembly by a Newly Identified SR Protein Kinase
Source: PLoS Biol. 2009 Sep 22;7(9):e1000193. doi: 10.1371/journal.pbio.1000193 (PMC2737616; doi:10.1371/journal.pbio.1000193)

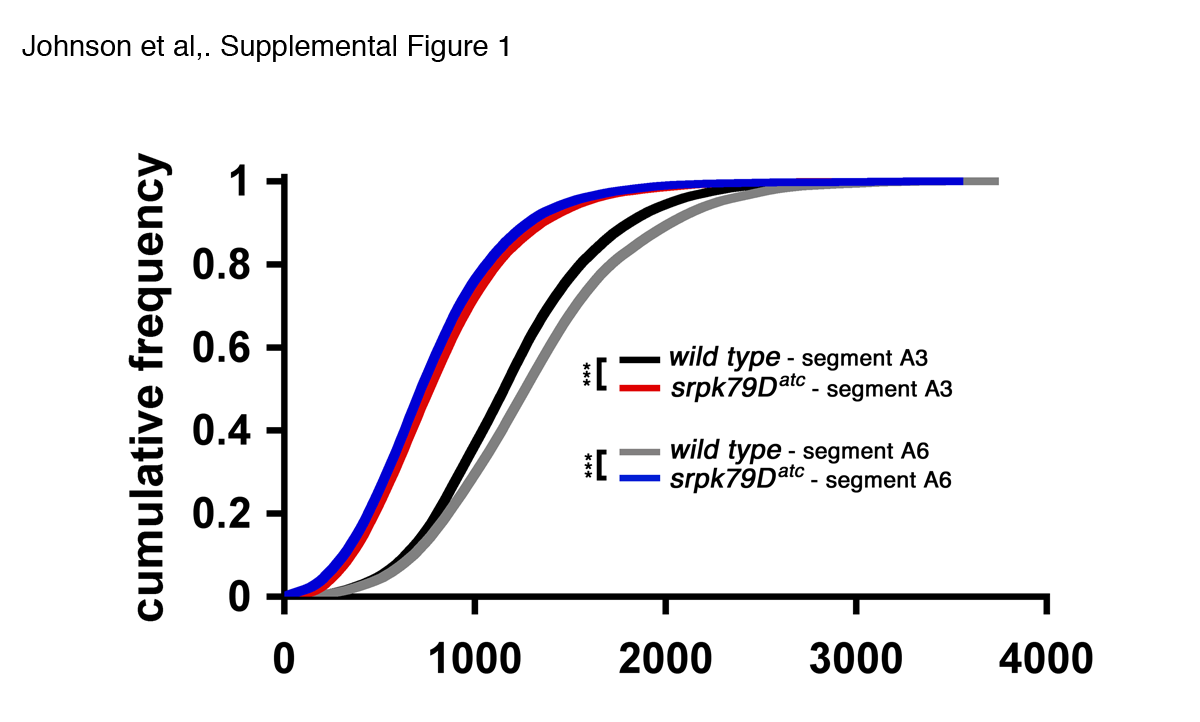

Supplement: Figure S1 — Analysis of synaptic Brp levels in anterior and posterior segments from wild type and srpk79Datc mutants. Cumulative frequency plots of individual muscle 4 NMJ Brp puncta maximum pixel intensities from wild-type abdominal segment A3 (black curve), srpk79Datc segment A3 (red curve), wild-type segment A6 (grey curve), and srpk79Datc segment A6 (blue curve). There is a slight, though statistically significant, increase in synaptic Brp puncta intensity in wild-type segment A6 (posterior) compared to A3 (anterior). In homozygous srpk79Datc mutants, there is a smaller difference comparing anterior and posterior segments. In conclusion, there is a large decrease in Brp synaptic intensity in the mutant compared to wild type, and this is not dramatically affected by the position of the NMJ along the anterior-posterior axis. Each curve represents data collected from synaptic arbors of a total of 36 synapses from 18 larvae. n = 4,657, 3,518, 6,134, and 4,638, respectively. * = p<0.05, ** = p<0.01, *** = p<0.001, Mann-Whitney U Test. (0.14 MB TIF) [file pbio.1000193.s001.tif]

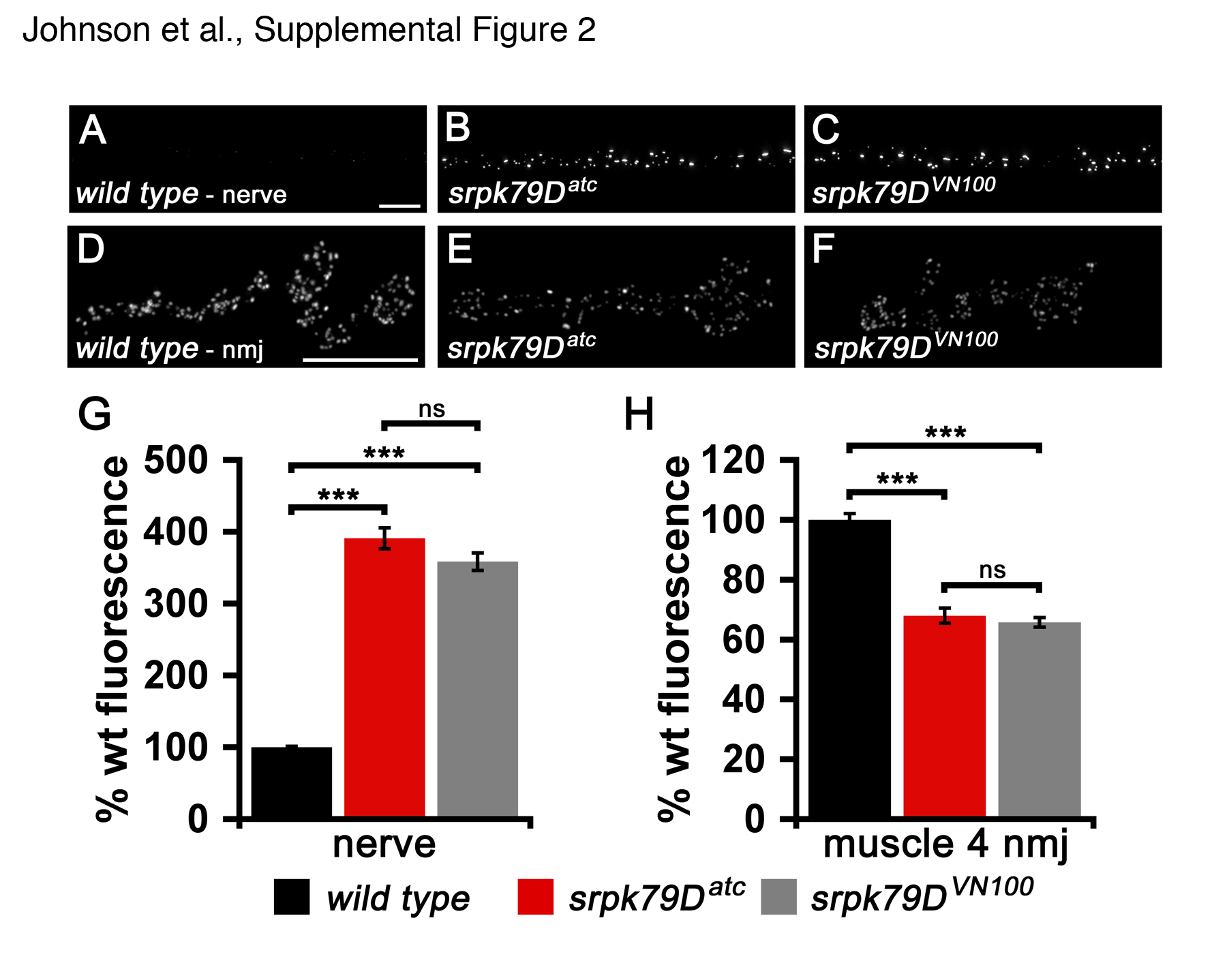

Supplement: Figure S2 — Analysis of an additional srpk79D mutation. (A–C) Representative immunofluorescence images of individual larval nerves stained with anti-Brp antibody from wild-type (A), srpk79Datc (B), and srpk79DVN100 (C) larvae. (D–F) Representative immunofluorescence images of muscle 4 NMJs stained with anti-Brp antibody from wild-type (D), srpk79Datc (E), and srpk79DVN100 (F) larvae. (G and H) Bar graphs showing average (±the standard error of the mean [SEM]) nerve (G) and muscle NMJ (H) staining intensities. The increases in nerve staining and decreases in synaptic NMJ in the two srpk79D mutant conditions are both highly statistically significant compared to wild type and statistically indistinguishable from each other. Each bar represents data taken from 24 synapses from 12 larvae. *** = p<0.001, Student t-test. Scale bars indicate 10 µm. (0.32 MB TIF) [file pbio.1000193.s002.tif]

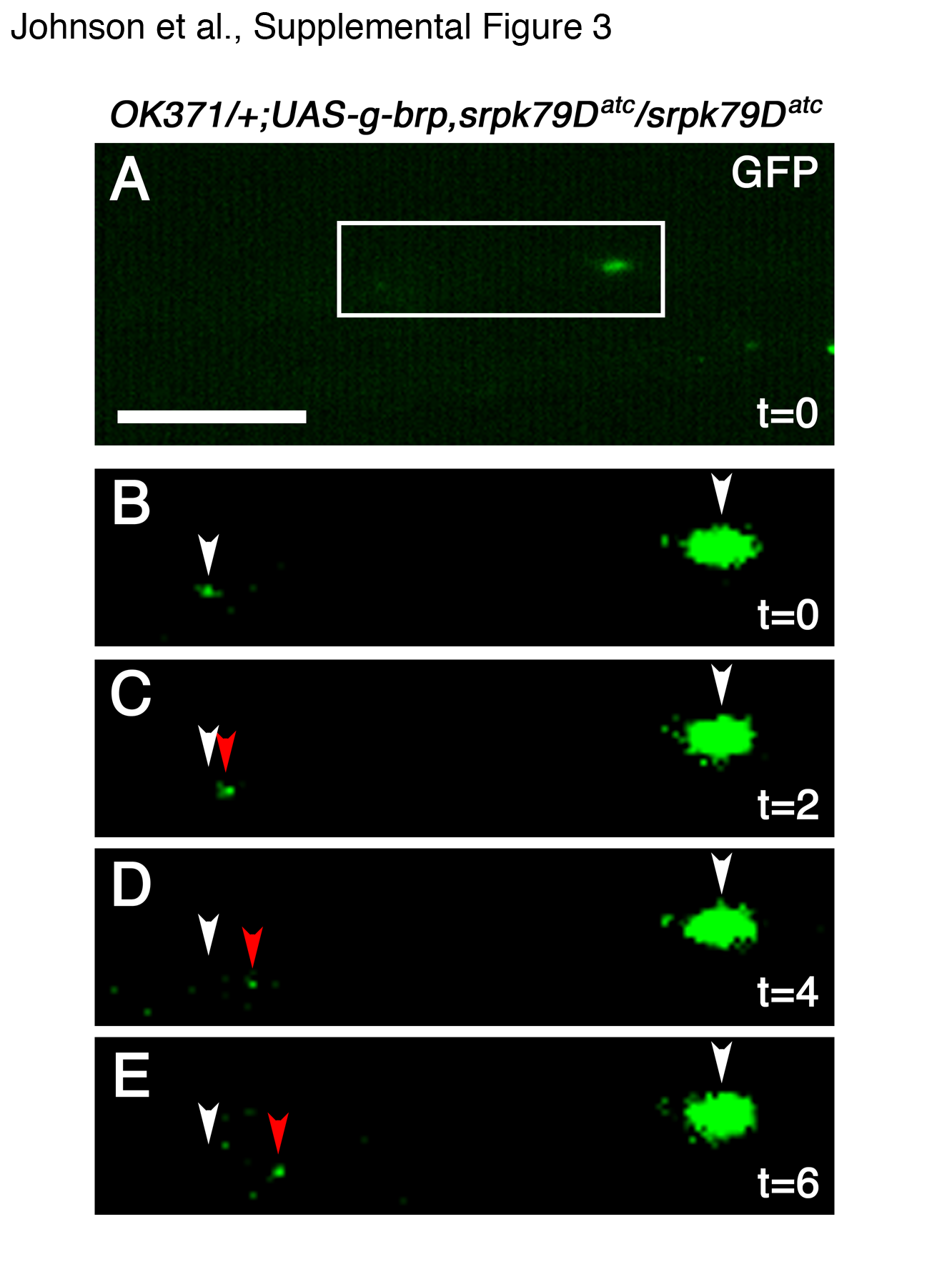

Supplement: Figure S3 — Live image analysis. (A) Live GFP fluorescence image of nerves from a srpk79Datc mutant larvae expressing GFP-tagged Brp in motoneurons at time = 0 s. Several small and large GFP-Brp puncta are apparent. Scale bar indicates 5 μm. (B–E) show a time series (t = 0 to t = 6 s) of upper-left boxed region in (A) demonstrating a motile small BRP punctum and an immobile large BRP aggregate. In (B–E), white arrows indicate the GFP-Brp punctum/aggregate position at the beginning of the time series. Red arrows highlight the new position of the motile punctum in each frame. In these experiments, we sought to directly assess Brp transport in srpk79Datc mutants through live imaging of srpk79Datc mutant larval nerves expressing a GFP-tagged brp cDNA in motoneurons [4] (OK371/+;UAS-g-brp, srpk79Datc/srpk79Datc). A similar method was used previously to observe GFP-tagged synaptotagmin transport [18]. In our experiments, a variety of GFP-Brp species were observed including small puncta and large accumulations. The majority of small GFP-Brp puncta were found to be motile with individual puncta exhibiting anterograde and/or retrograde transport during a given imaging epoch. The average rate of small GFP-Brp puncta transport was 0.08±0.009 µm/s (average±SEM). Although this rate is somewhat slower than values previously reported for the transport of GFP-tagged Syt, it falls within the range of reported values [18]. In contrast, large GFP-Brp accumulations were generally immotile (as shown here). Occasionally, a large accumulation was observed to alternately exhibit anterograde and then retrograde transport. However, total displacement was never more than 0.3 µm (unpublished data). (0.43 MB TIF) [file pbio.1000193.s003.tif]

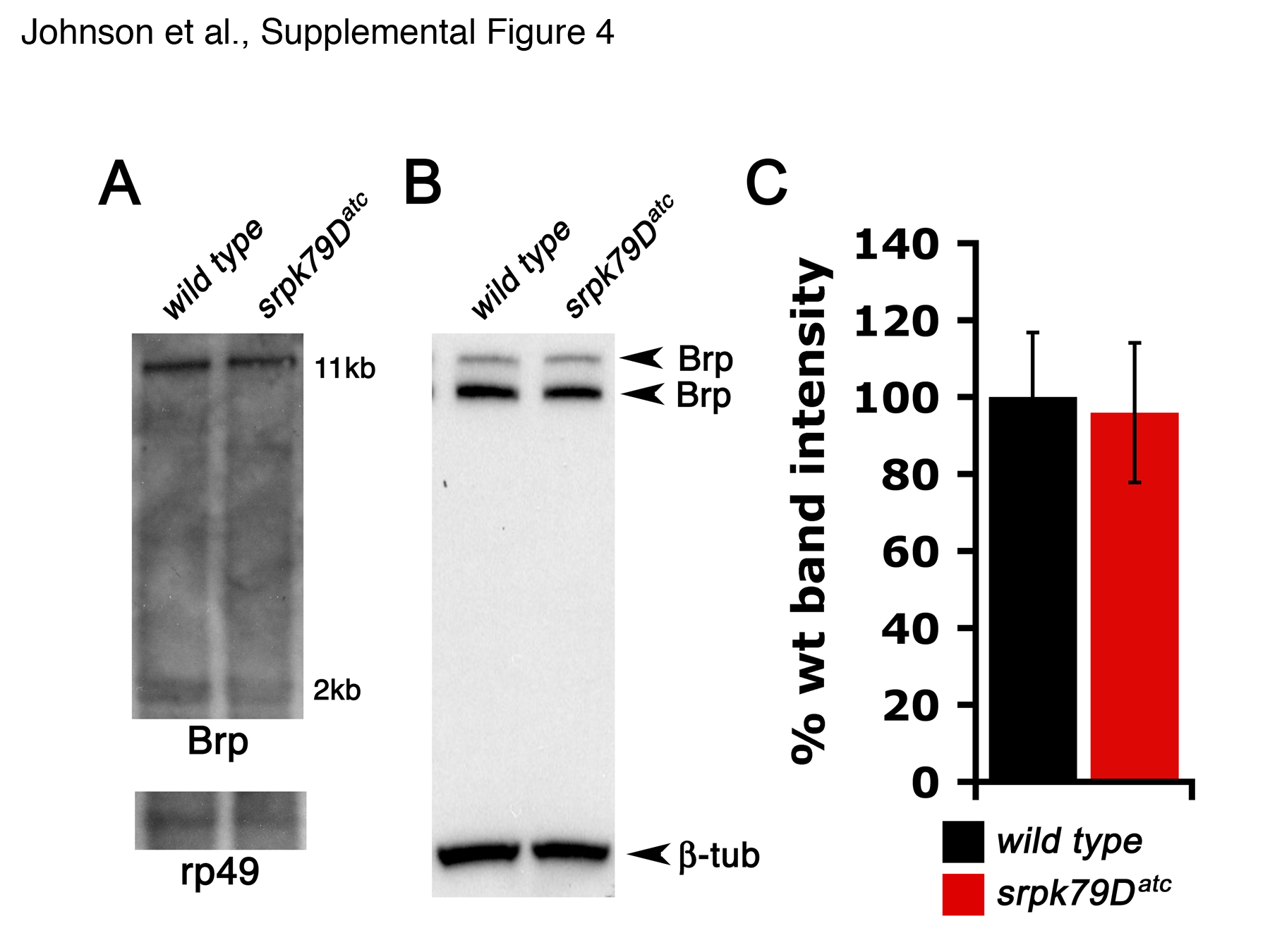

Supplement: Figure S4 — Supplemental molecular analyses. (A) Northern blot analysis of Brp transcripts in mRNA purified from wild-type (left lane) or homozygous srpk79Datc mutant (right lane) adult fly heads indicates no difference in mRNA processing between the two genotypes. Approximately 10 µg of mRNA was loaded in each lane. An RNA sequence complementary to a region of the Brp mRNA found in all known transcripts was used to probe the blot. The lower panel shows rp49 mRNA signal as a loading control. (B) Example western blot of protein extracted from wild-type (left lane) or homozygous srpk79Datc mutant (right lane) larval CNSs. There is no detectable change in Brp expression levels. Bar graph showing average (±SEM) wild-type and srpk79Datc anti-Brp band intensities (normalized to anti-β-tubulin band intensity) shows that Brp is not overexpressed as a consequence of srpk79D loss of function. Bars represent data taken from six independent experiments. (0.51 MB TIF) [file pbio.1000193.s004.tif]

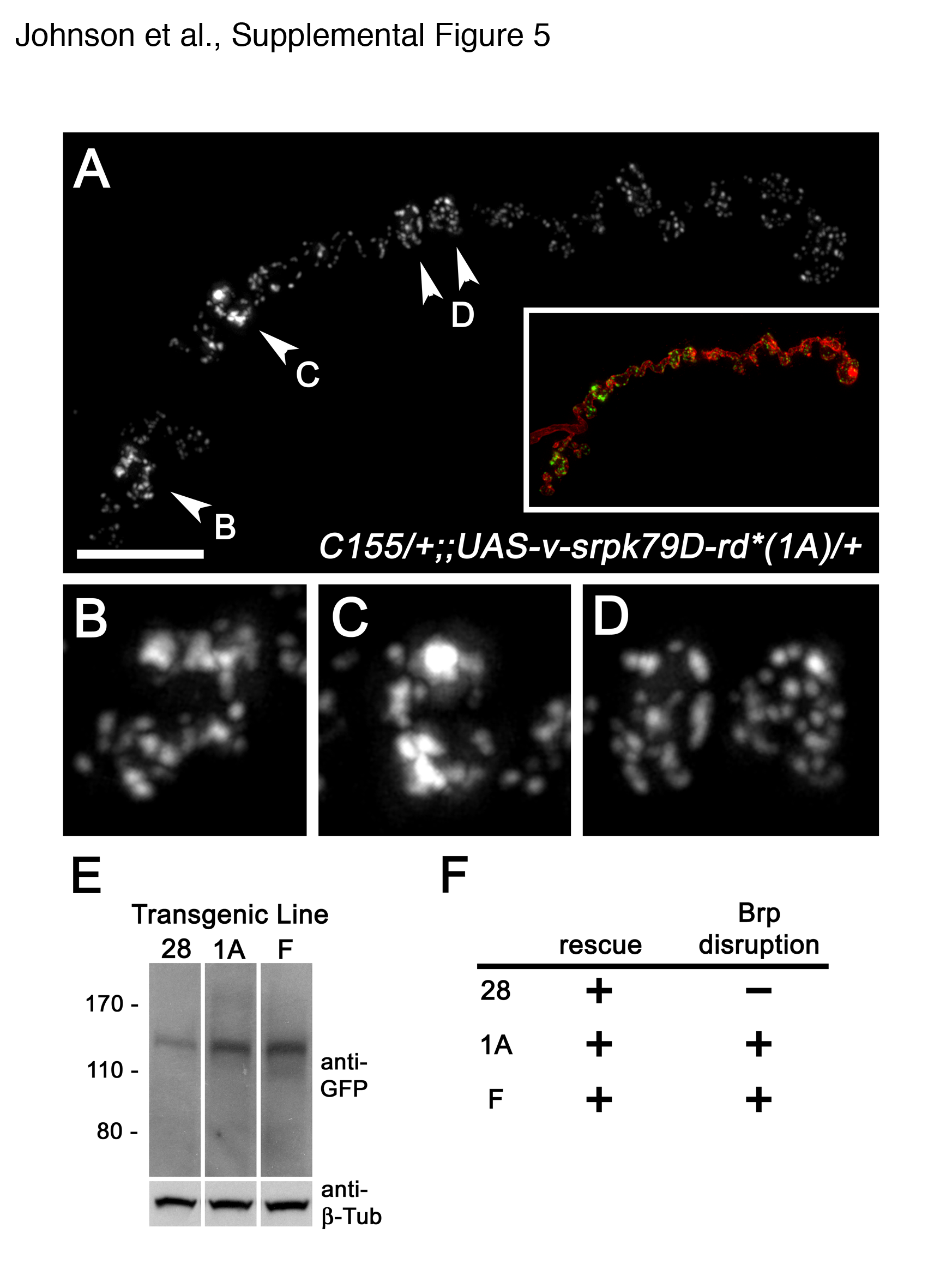

Supplement: Figure S5 — Overexpression of SRPK79D-RD* causes synaptic Brp disruption. (A) Example anti-Brp immunofluorescence image of a muscle 4 NMJ from an animal expressing high levels of SRPK79D-RD*. Inset shows the same synapse including both anti-Brp immunofluorescence (green) and anti-HRP immunofluorescence (red) channels for orientation. Arrowheads highlight boutons with disrupted anti-Brp staining, which are magnified in (B–D). (B–D) Magnified images of highlighted boutons in (A). (E) Western blot analysis of protein extracted from three independent UAS-v-srpk79D-rd* transgenic lines showing differential protein expression. (F) Table summarizing the ability of each transgenic line in (E) to rescue the srpk79D mutant phenotypes and to disrupt Brp organization. Scale bar indicates 10 µm. (0.74 MB TIF) [file pbio.1000193.s005.tif]
