# Supplementary material for: Negative Regulation of Active Zone Assembly by a Newly Identified SR Protein Kinase
Source: PLoS Biol. 2009 Sep 22;7(9):e1000193. doi: 10.1371/journal.pbio.1000193 (PMC2737616; doi:10.1371/journal.pbio.1000193)
Supplement: Text S1 — Supplemental discussion relevant to Figure 8 . (0.03 MB DOC) [file pbio.1000193.s006.doc]

**Supplemental Discussion Relevant to Figure 8.**

Two transcripts derived from the *srpk79D* locus were cloned previously by Stapelton et al. (2002) as part of the Berkeley Drosophila Genome Project [62]. Early in our study we noticed that the cloned cDNA from the second transcript (*srpk79D-rd*) differed from the predicted splicing pattern and would lead to the production of a protein product with a truncated kinase domain. Subsequently, we used RT-PCR to confirm the existence of a transcript that conformed to the predicted splicing pattern (srpk79D-rd*) and generated transgenic lines that express this transgene under GAL4-UAS control. This transgene rescues the Brp accumulation phenotype (Fig. 3G-3J). Thus, we are confident that srpk79D-rd* is produced from the *srpk79D* locus and is biologically relevant.
